# Analysis of expression in the *Anopheles gambiae *developing testes reveals rapidly evolving lineage-specific genes in mosquitoes

**DOI:** 10.1186/1471-2164-10-300

**Published:** 2009-07-06

**Authors:** Elzbieta Krzywinska, Jaroslaw Krzywinski

**Affiliations:** 1 Department of Biology, University of Texas at Arlington, Texas 76019, USA; 2 Vector Group, Liverpool School of Tropical Medicine, Liverpool L3 5QA, UK

## Abstract

**Background:**

Male mosquitoes do not feed on blood and are not involved in delivery of pathogens to humans. Consequently, they are seldom the subjects of research, which results in a very poor understanding of their biology. To gain insights into male developmental processes we sought to identify genes transcribed exclusively in the reproductive tissues of male *Anopheles gambiae *pupae.

**Results:**

Using a cDNA subtraction strategy, five male-specifically or highly male-biased expressed genes were isolated, four of which remain unannotated in the *An. gambiae *genome. Spatial and temporal expression patterns suggest that each of these genes is involved in the mid-late stages of spermatogenesis. Their sequences are rapidly evolving; however, two genes possess clear homologs in a wide range of taxa and one of these probably acts in a sperm motility control mechanism conserved in many organisms, including humans. The other three genes have no match to sequences from non-mosquito taxa, thus can be regarded as orphans. RNA *in situ *hybridization demonstrated that one of the orphans is transcribed in spermatids, which suggests its involvement in sperm maturation. Two other orphans have unknown functions. Expression analysis of orthologs of all five genes indicated that male-biased transcription was not conserved in the majority of cases in *Aedes *and *Culex*.

**Conclusion:**

Discovery of testis-expressed orphan genes in mosquitoes opens new prospects for the development of innovative control methods. The orphan encoded proteins may represent unique targets of selective anti-mosquito sterilizing agents that will not affect non-target organisms.

## Background

Mosquitoes transmit devastating infectious diseases that kill up to three million people and debilitate hundreds of millions every year [1]. Large-scale control campaigns have been attempted to lessen this enormous burden, but despite initial promise they have become increasingly inefficient – largely because of emergence and spread of drug resistance in pathogens and insecticide resistance in the mosquito vectors. As a result, the numbers of cases of mosquito-borne diseases is rising [2,3], which points to an urgent need to devise more effective control strategies. Developing novel mosquito-based approaches requires an extensive knowledge of mosquito biology. The ecology, population genetics, molecular biology, and genomics of *Anopheles gambiae *and *Aedes aegypti*, major vectors of malaria and yellow fever/dengue, respectively, have been intensively studied in recent years, which culminated in sequencing of their genomes [4,5]. However, many aspects of their biology relevant to control efforts remain poorly understood.

The life cycle of vector mosquitoes depends on a protein-rich bloodmeal required for egg development, and repeated blood feeding assists in the transmission of pathogens. Because only female mosquitoes are involved in these processes, laboratory research efforts have been directed almost exclusively at understanding the female physiology and molecular biology, with the goal of identifying features that may be used in innovative interventions to fight the diseases [6]. However, when control methods, aiming either at reduction of mosquito populations or population replacement, are considered, males cannot be ignored and their characteristics, including male-specific transcripts and proteins should also be viewed as potential targets. In this respect, genes expressed in male reproductive organs are of particular interest for three reasons. First, their protein products may constitute suitable targets of novel classes of agents that would cause male sterility. Theoretically, male-sterilizing insecticides may be superior to the currently used ones. In several animal groups, including insects, male-expressed genes evolve significantly faster than female-biased genes, or those with sex-unbiased expression [7], and a number of genes expressed in testes have been hypothesized to originate in recently evolved lineages [8,9]. Therefore, insecticides affecting proteins involved in spermatogenesis may target exclusively mosquitoes and be harmless to non-target organisms. Moreover, mutations causing resistance to such insecticides would probably seriously affect fertility and individuals carrying the mutations would be quickly eliminated from the population. Second, *cis*-regulatory sequences of genes expressed in male germline may be used in genetic control strategies as elements of gene drive system to spread desired characteristics, such as resistance to pathogens, into wild mosquito populations. Several driving systems, based on activity of selfish genetic elements, have been proposed [10] and major advances have been made in the creation of modified transposons and homing endonucleases [11]. However, to be spread to subsequent generations, such selfish elements must be activated in the germ line; moreover, they should be restricted to these tissues to limit the mutagenic effects of transposition in soma, which may reduce the fitness of transgenic mosquitoes [12]. Two genes, *oskar *and *nanos*, expressed in the female germ line were proposed as candidates for donating regulatory sequences to control modified transposons in mosquitoes [12,13], and the regulatory elements of *nanos *have been demonstrated to mediate transposition in *A. aegypti *in a developmentally regulated manner [14]. Homing endonucleases have never been found in animals; however, the activity of fungus-derived enzymes have recently been reported in *An. gambiae *[11,15]. The potential of driving transgenes using male germ line promoters remains to be tested, but any such attempt is hampered by inadequate information about testis-specific expression patterns. Third, such genes may be targeted to induce male sterility in an improved sterile insect technique [16].

Several microarray analyses explored differential gene expression between adult female and male mosquitoes, providing a genome-wide overview of male-biased expression [5,17-19]. Moreover, *in silico *analysis identified a number of genes expressed in the adult male accessory glands [20]. However, information about gene expression in testes is almost completely lacking, the only exception being the analysis of *β2 tubulin *gene, earlier identified to be testes-specific in *Drosophila *[21], and found to have conserved testes expression in *Aedes aegypti *[22], *An. gambiae *[11], and *Anopheles stephensi *[23].

To gain a better understanding of gene expression in males we targeted transcripts from the reproductive tissues of *Anopheles gambiae *male pupae. Using a cDNA subtraction strategy we identified five genes, whose spatial and temporal patterns of transcription suggest involvement in the late phases of spermatogenesis. Comparative genomic analysis revealed that three of these genes have homologs detectable only in mosquitoes, suggesting that they have a rapid evolutionary rate or have a relatively recent origin in the mosquito lineage. The two other genes have homologs in a wide range of taxa, with which they share short conserved sequence regions; one of these genes may have a highly conserved function in controlling sperm flagellum motility. In addition to *in silico *sequence analyses, we conducted an analysis of ortholog expression in three mosquito species and discovered substantial differences in expression profiles of three genes, which in *Aedes *and *Culex *are expressed in a sex-unbiased pattern.

## Results and Discussion

### Isolation of male-specific transcripts by suppressive subtractive hybridization

Enrichment of male transcripts was done using a suppressive subtractive hybridization (SSH) strategy, in which fragmented male cDNA was ligated to adaptor sequences and subjected to liquid hybridization with an excess of female cDNA. Subsequently, a selective amplification of the male-specific cDNA was performed in two rounds of PCR using external and nested primers complementary to the adaptors. The second round of PCR performed using nested primers yielded 23 fragments, ranging in size from 200 bp to > 2 kb, discernible on a smeary background upon fractionation on an agarose gel (data not shown). Each fragment was excised from the gel, cloned, and sequenced. The sequences were subjected to BLASTN searches against NCBI's *Anopheles gambiae *EST collection to eliminate transcripts known to be expressed in female tissues. The sequences that had no match to female ESTs were tested by RT-PCR for male-specific expression using pupal male and female total RNAs as a template. Five sequences showed strong expression in male pupae and were analyzed further (see Additional file 1 for details on sequences not included in further analysis).

The SSH strategy implemented yields fragments of cDNAs. The full-length sequences were assembled following identification of the transcripts' ends using 5' and 3' RACE and found to correspond to five different genes. Four of those do not match any *An. gambiae *gene predictions and only a fragment of one gene has previously been predicted to encode an *An. gambiae *protein (VectorBase *An. gambiae *gene build AgamP3.4). Basic sequence characteristics of each gene are presented in Table 1; their cDNA sequences are available in GenBank under accession nos. FJ869232–FJ869243. The putative full-length protein-coding regions were used as queries in TBLASTN searches for their homologs in other organisms. Orthologous sequences of all five genes were identified in the *Aedes aegypti *and *Culex quinquefasciatus *genomes. Two of those genes also have putative orthologs in a wide range of taxa, including humans, in which they encode poorly characterized or uncharacterized proteins; here we name them *AgRopn1l *and *AgDzip1l*, after their putative human orthologs. Three genes, named here *mts *(mosquito testis specific), *Ams *(*Anopheles *male specific) and *AAms *(*Anopheles *and *Aedes *male *s*pecific), code for proteins that exhibit no similarity to any GenBank entry derived from non-mosquito taxa, and as such they are regarded as orphan genes. They may have originated in the mosquito lineage or have been under a strong positive selection resulting in a rapid evolutionary rate and divergence from their non-mosquito orthologs beyond detection using the TBLASTN search.

**Table 1:** Basic sequence characteristics of the *Anopheles gambiae *genes isolated during this study and their orthologs from *Aedes aegypti *and *Culex quinquefasciatus*.

Species	Length (nt)	5'UTR (nt)	Coding region (nt)	3'UTR (nt)	Protein length (aa)	Identity//similarity^1^ (%)	Gene status^2^
***AgRopn1l***							
*An. gambiae*	2391–2506^3^	29–144^3^	2040	319	679		unannotated
*Ae. aegypti*			2166		721	54.2/65.3	**14993**
*Cx. quinquefasciatus*			2091		696	74.7/83.8	*07290*
***AgDzip1l***							
*An. gambiae*	2623–2707^3^	76–160^3^	2547	48	848		*01165*
*Ae. aegypti*			2502		833	46.5/58.1	unannotated
*Cx. quinquefasciatus*			2328		775	63.5/74.2	*11569*
***Ams***							
*An. gambiae*	1356	39	1227	90	408		unannotated
*Ae. aegypti*			nd^4^		nd	13.8/30.8^5^	unannotated
*Cx. quinquefasciatus*			nd		nd	70.8/89.9^5^	unannotated
***mts***							
*An. gambiae*	1122	108	897	117	295		unannotated
*Ae. aegypti*			1102		346	25.1/36.1	*09638*
*Cx. quinquefasciatus*			912		303	45.8/54.9	**00571**
***AAms***							
*An. gambiae*	3724	87	3567	157	1188		unannotated
*Ae. aegypti*			4707		1568	22.6/38.5	unannotated
*Cx. quinquefasciatus*			5022		1673	36.1/50.4	unannotated

^1 ^Protein sequence identity/similarity calculated after exclusion of gapped regions. Values for the *An. gambiae*/*Ae. aegypti *comparisons are given in the rows corresponding to *Aedes aegypti*; these values are also representative of the identity/similarity between *An. gambiae *and *Cx. quinquefasciatus*. The identity/similarity between *Ae. aegypti *and *Cx. quinquefasciatus *proteins is given in the *Cx. quinquefasciatus *rows.

^2 ^The Ensembl gene ID (abridged by omitting the species codes AGAP0, AAEL0, and CPIJ0 for *An. gambiae*, *Ae. aegypti*, and *Cx. quinquefasciatus*; respectively) is given for genes with complete (bold) or partially (italic) predicted sequence in the recent VectorBase gene builds (*An. gambiae*: AgamP3.4; *Ae. aegypti*: AaegL1.1; *Cx. quinquefasciatus*: CpipJ1.0_5); genes that do not match any gene predictions are marked as unannotated.

^3 ^Several alternative transcription start sites identified.

^4 ^nd – not determined

^5 ^Data based on a portion of a gene sequence; see text for details.

### Expression profiling of male-specific genes

**Figure 1:**
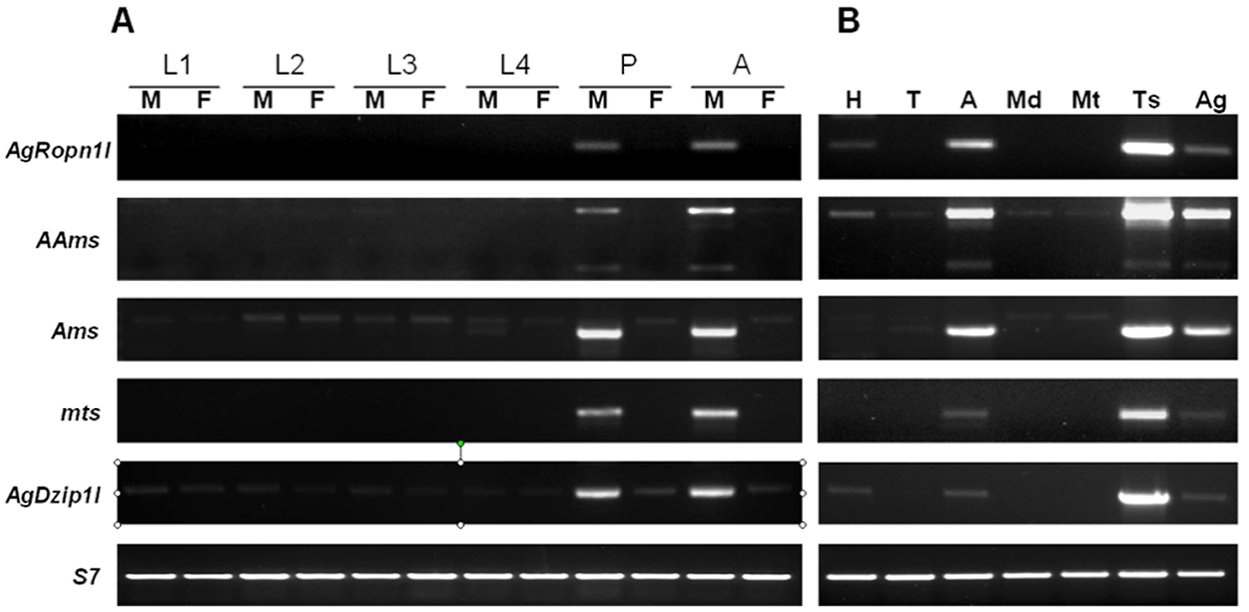
**RT-PCR analysis of expression of the *Anopheles gambiae *genes identified in this study**. (A) Expression during the course of development. M, male; F, female; L1, first instar larvae; L2, second instar larvae; L3, third instar larvae; L4, fourth instar larvae; P, pupae; A, adults. (B) Male tissue expression. H, head; T, thorax; A, abdomen; Md, midgut; Mt, Malpighian tubules; Ts, testes; Ag, accessory glands. Apart from *AAms*, the primers used for RT-PCR were designed to span intron regions, which allows an easy recognition of the RT-PCR signal from the amplified traces of genomic DNA contaminating the RNA samples despite DNase treatment (cf. *Ams *products, in which the weak upper band represents the DNA amplified from a genomic template and the lower band corresponds to cDNA fragmens). The low level of signal in *AAms *likely originates from residues of genomic DNA, because the amplified fragment spans an exclusively exonic region. For each sample 28 amplification cycles were performed, apart from 32 cycles for *AAms *in dissected tissues to visualize the short transcript. *S7 *was amplified in 24 cycles.

The temporal profile of gene expression was evaluated by RT-PCR using total RNA templates isolated separately from male and female *An. gambiae *sampled from each postembryonic developmental stage. For each gene the expression was practically limited to male pupae and adults (Figure 1A). There was comparable mRNA abundance in pupae and adults or a slight increase was observed in two-day-old adult males and subsequently maintained at that level until at least 10 days post emergence (data not shown). An evident departure from this pattern was found in the *AgDzip1l *profile, in which a low level of expression was ubiquitously present in male larvae and all female stages in addition to a strong expression in pupae and adult males. Moreover, an earlier onset of transcription, during the fourth larval instar, was detected for the *Ams *gene (cf. a faint band in a L4 male lane, Figure 1A).

**Figure 2:**
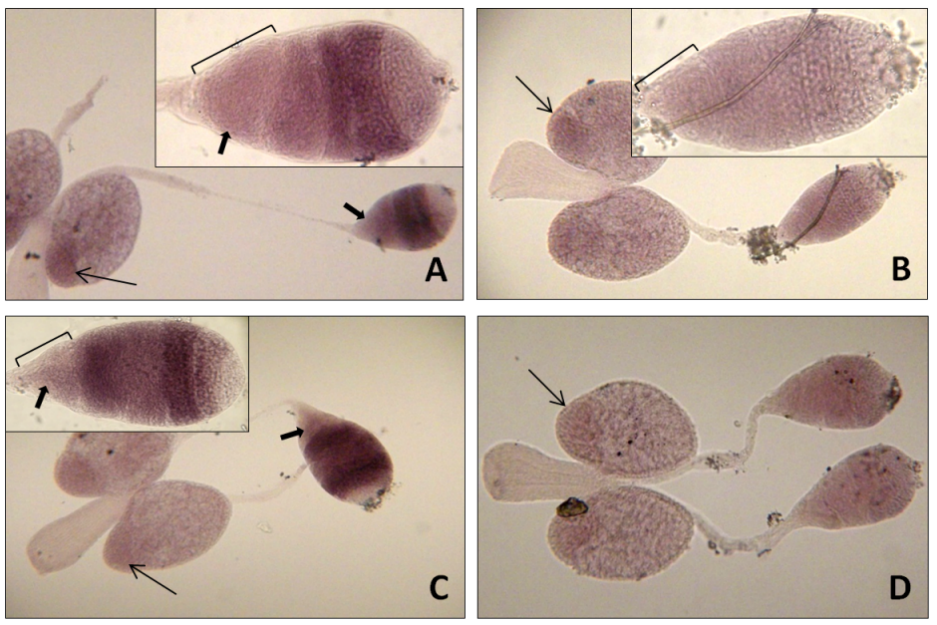
**RNA *in situ *hybridization analysis of expression of the *Ams *and *mts *genes in the *An. gambiae *male reproductive tissues**. (A) Hybridization of the *Ams *antisense probe reveals that abundant transcripts are present in primary and secondary spermatocytes; afterwards the transcripts are gradually degraded, but persist at low level in spermatozoa (small solid arrow). (B) Hybridization of the sense *Ams *probe produces no signal. (C) Hybridization of the *mts *antisense probe shows active transcription of the gene in primary spermatocytes and spermatids, and persistence of the transcripts in spermatozoa (small solid arrow). (D) Hybridization of the *mts *sense probe produces no signal. On each photograph testes are located on the right and accesory glands with ejaculatory duct on the left. Within testis germ cells of the same developmental phase are encased in a single cyst. Unstained apical part of each testis, containing the youngest germ cells, points to the right. The leftmost compartment (encompassed by brackets in insets) represents a sperm reservoir with mature spermatozoa. The low background seen in all samples originates from the color reaction induced by endogenous lysosomal alkaline phosphatases. Note that a stronger background in the accessory gland basal lobes, containing denser granular tissue (arrow), is present in all samples, regardless of the probe used for hybridization.

The spatial expression pattern was analyzed using RT-PCR and, for two selected genes (*Ams *and *mts*), using whole-mount RNA *in situ *hybridization to the male reproductive tissues. Total RNA from the undissected head, thorax and abdomen, and dissected abdominal tissues of adult males served as templates for RT-PCR experiments. Relatively abundant transcripts of each gene were detected in the abdomen, where most of the expression was apparently limited to the testes (Figure 1B). Various amounts of RT-PCR products were also detected in the accessory gland samples. *In situ *experiments confirmed the localization of the *Ams *and *mts *transcripts in the testes, however, no hybridization signal was evident in the accessory glands (Figure 2); this hybridization pattern likely holds for the other three genes not examined using RNA *in situ *method. While a low level of transcription in the gland tissue cannot be excluded (note a relatively high background in the *in situ *experiments), it is possible that the RT-PCR product in those samples is an experimental artifact, arising from the reproductive tissue dissection method. First, some testis RNA carry-over to the accessory gland samples could have occurred during dissection. Second, because the accessory glands were excised with fragments of *vasa deferentia *and seminal vesicles, which in adult males may contain mature sperm, it is likely that the transcripts detected in the accessory gland RNA samples have actually originated from the spermatozoa. Indeed, for both genes tested by *in situ *hybridization, a faint signal is present in the testis sperm reservoir (Figure 2A, C; Additional file 1). The retention of a complex mRNA population in the ejaculated sperm is well documented in humans, but is known to occur in the male gametes of many species [24]. We assume that the expression detected in the late preimaginal stages is, as in adult males, limited to the reproductive tissues.

The localization and timing of transcription suggests that the identified genes are involved in the mid-late phases of spermatogenesis. Spermatogenesis proceeds in succession from primordial germ cells differentiated in the embryo, through primary and secondary spermatogonia, primary and secondary spermatocytes, to spermatids and, ultimately, spermatozoa [25]. The last step in this process, called spermiogenesis and involving cytodifferentiation of round spermatids into long mature spermatozoa, is particularly complex. In mosquitoes spermiogenesis is thought to occur mainly during the pupal stage, but considerable temporal variation of this process exists in different mosquito groups [25]. In *Anopheles*, spermatogenesis progression has been described in the fourth instar larvae, pupae and newly emerged adults of a European species *An. atroparvus *(subgenus *Anopheles*) [26] and, indirectly, in adults of *An. culicifacies *[27], *An. gambiae *[28], and *An. stephensi *[29] (all three from subgenus *Cellia*). In *An. atroparvus *spermatogenesis progression is fast, with the first spermatozoa observed in the 96 hours old fourth instar larvae [26]. All phases of spermatogenesis still occur in young pupae, in which early germ cells occupy the apical part and maturing spermatozoa the posterior part of the testis. In late pupae and newly emerged adults most of the testicular cell population is represented by mature sperm bundles. In contrast, in representatives of *Cellia *spermatogenesis continues well into the adult stage. In newly emerged males over half of the testis is occupied by spermatocysts containing immature germ cells at different stages of development. With age, posterior-most spermatocysts mature and rupture to release spermatozoa, resulting in an increasing proportion of testis occupied by the sperm reservoir. In virgin males the rate of spermatogenesis progressively slows down, however, mating-associated sperm depletion leads to an increase in the number of spermatocysts, indicative of a capacity of adults to rapidly resume spermatogenesis [29]. Age-related changes in the numbers of spermatozoa have not been studied in *Anopheles*, but in virgin males of *Ae. aegypti *the sperm count increases more than two-fold between day 1 and day 10 after eclosion [30].

To date, *β2 tubulin *remains the only gene reported to be testis-specific in *An. gambiae*. Similar to *Drosophila β2 tubulin *[21], it presumably functions in meiotic spindle formation, sperm cell shaping, and axoneme assembly, and is activated in primary spermatocytes during the late third instar larval stage [11]. The genes identified in our study are activated in fourth instar larvae or pupae, but their expression is also initiated in the (late) primary spermatocytes, as demonstrated by RNA *in situ *hybridization to *Ams *and *mts *transcripts (Figure 2). Interestingly, hybridization patterns of these two transcripts differ, indicating involvement of each gene in different spermatogenic processes. *Ams *transcript abundance persists unchanged in secondary spermatocytes and is later gradually reduced. The *mts *transcripts are partially eliminated in the secondary spermatocyte stage, but, unexpectedly, their level is significantly increased in spermatids. It is generally believed that in *Drosophila *genes required during meiosis and all post-meiotic spermatogenic processes are transcribed exclusively in primary spermatocytes and stored in the cytoplasm for later use. However, transcription of 24 genes has been recently detected in the fly's spermatids [31], similar to the *mts *gene.

A relatively late onset of expression with respect to the spermatogenic program suggests that the promoters of the identified genes may not be optimal for driving transgenes in genetic mosquito control strategies. However, absence of data on gene regulation in mosquito testes prompted us to search for the putative testis-specific *cis-*regulatory elements. For this purpose we used the pattern discovery tool from RSAT program to identify overrepresented hexa-, hepta- and octamer motifs in the 2 kb regions upstream from the putative translation start sites of the five genes. No significantly overrepresented motifs common to all sequences were found, however, 5' flanks of four genes contained a CGTTGAA heptamer. Its significance remains to be tested, but it should be noted that a putative regulatory motif with similar sequence (ACGTTGA) has been identified in a screen of upstream regions of all orthologous genes annotated in *Anopheles*, *Aedes *and *Culex *genomes [32].

Intriguingly, the *AgRopn1l*, *AgDzip1l*, and *AAms *were also transcribed at low levels in the head. The significance of this observation remains unknown, but it is tempting to speculate that their products may be involved in modulation of adult male physiology and behavior, such as female seeking and mating. Finally, *Ams *was expressed at very low levels in the head and thorax.

### Comparison of orthologues from other mosquitoes

**Figure 3:**
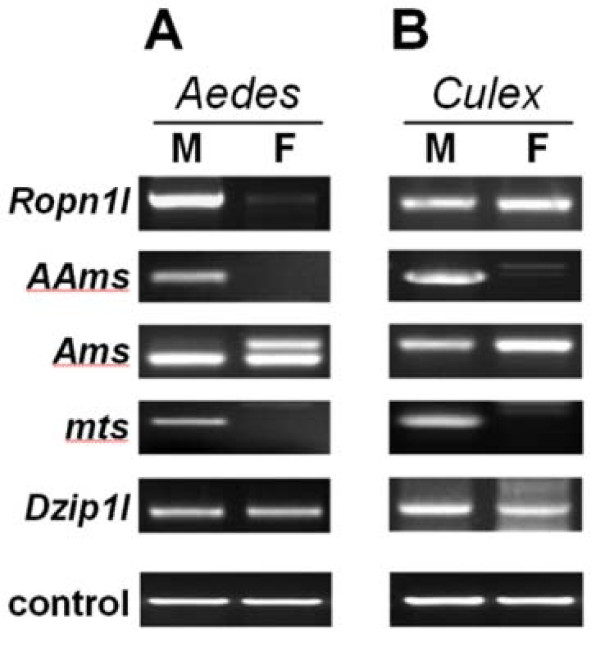
**RT-PCR analysis of expression of the *Aedes aegypti *and *Culex quinquefasciatus *orthologs to the *Anopheles gambiae *genes identified in this study**. (A) Expression in *Aedes aegypti*. The *Ams *ortholog yields two transcripts differing in size due to an intron splicing; intron retention in a female (the upper band) results in a transcript encoding a truncated protein. Similarly, the retained intron leading to protein truncation is present in both sexes in *Culex*. See Supplementary Figure 3 for further details. (B) Expression in *Culex quinquefasciatus*. The *Ams *ortholog yields a single transcript corresponding to the *Ae. aegypti Ams *transcript with an intron retained. M, male; F, female. In female lanes, faint bands differing in size from the male bands represent non-specific amplification products. *Actin-1 *gene and ribosomal *S7 *gene were used as a control of equal sample loading in *Ae. aegypti *and *Cx. quinquefasciatus*, respectively.

Using RT-PCR, we explored the expression patterns of all *An. gambiae *orthologs in both sexes of *Ae. aegypti *and *Cx. quinquefasciatus *pupae and adults. All orthologs were expressed in males of both species, but only the *mts *gene was apparently expressed in males exclusively (Figure 3, Additional file 1). The other genes were expressed in *Aedes *and *Culex *females at various levels, ranging from very low (with strong male bias) to high (with sex-unbiased or female-biased expression). The latter cases, concerning the *Ropn1l *gene in *Culex*, the *Dzip1l *gene in *Aedes*, and the *Ams *orthologs in both *Aedes *and *Culex*, indicate either additional functions for these genes in females or different functions in these species altogether. Contrary to our study a microarray-based comparison of gene expression in *Ae. aegypti *adults showed no male bias in *Ropn1l *and *mts *transcript levels [5]. This discrepancy probably results from a lower sensitivity of microarrays as compared to RT-PCR in detecting differential gene expression. Our observations of expression divergence between *Anopheles *and culicine orthologs are not surprising in the light of *Drosophila *studies, which showed that male-biased genes are particularly prone to shifts in expression patterns leading to a gain, loss, increase, decrease, or reversal of sex-biased expression even in closely related species [33]. Such changes may be driven by sex-dependent natural selection, as suggested by significantly greater ratios of interspecific expression divergence to intraspecific expression polymorphism among male-biased genes compared to female-biased genes, or genes with sexually monomorphic expression patterns [34].

We searched the NCBI EST collection for mosquito expressed sequence tags matching the identified genes to provide additional evidence for their transcription (see the Additional file 1 for details). The transcripts were very poorly represented in the database. Only one out of ~153,000 *An. gambiae *ESTs unambiguously matched a fragment of one gene, 18 out of ~300,000 *Ae. aegypti *ESTs matched fragments of the three genes, and none of ~205,000 ESTs from *Cx. quinquefasciatus *were found to correspond to the identified genes.

Individual genes identified in the present study are described in more detail below.

### AgROPN1L

The *AgRopn1l *exhibits the highest degree of sequence and structure conservation among all the identified genes. Its encoded protein shares 65% similarity with *Aedes *and *Culex *orthologs (Table 1, Additional file 2), and 47% similarity with the human putative ortholog, ropporin 1-like protein (ROPN1L; known also as ASP). ROPN1L has been shown to interact with the sperm-specific A-kinase anchoring protein, AKAP3, via the amphipathic α helix region of AKAP3 [35]. AKAPs are a family of structurally diverse proteins that in somatic cells bind through this α helix domain to protein kinase A (PKA) after activation of the cAMP signal transduction pathway [36,37]. Human sperm-specific AKAPs are crucial to regulation of sperm motility and their function apparently relies on the interaction with ROPN1L [35]. Remarkably, ROPN1L shares high sequence similarity with the PKA region, known to form the groove necessary for docking to the amphipathic α helix of AKAPs [38].

**Figure 4:**
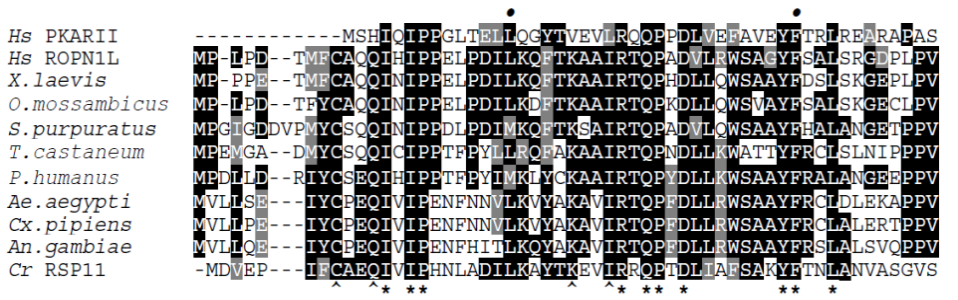
**Sequence alignment of the AKAP binding domain of the human PKA type II regulatory subunit with selected putative homologs of the ROPN1L protein**. Asterisks denote perfectly conserved positions across all sequences and caret (^) signs show additional positions conserved in ROPN1L homologs. Two dots above the alignment mark amino acid positions known to be vital for binding to AKAPs.

The *AgRopn1l *orthologs are widely distributed across the animal kingdom. Apart from human they were identified in other vertebrates, most insects, a sea urchin *Strongylocentrotus purpuratus*, a blood-fluke *Schistosoma japonicum*, and an organism as distant as the sea anemone *Nematostella vectensis*. In addition, a protein homolog of ROPN1L (called RSP11) was found within the radial spokes of the axonemal complex in a unicellular flagellate alga *Chlamydomonas reinhardtii*. A recent study showed that RSP11-deficient *Chlamydomonas *mutants display abnormal motility [39], which strongly suggests that the ROPN1L homologs constitute a critical component of the machinery controlling function of the flagella in a very wide range of organisms. However, the low level of *AgRopn1l *expression in the head implies an additional function of the protein in mosquitoes. All ROPN1L homologs are highly similar to each other and to PKA at the N terminus, where perfectly conserved positions mark residues apparently vital to the correct groove formation and binding to AKAPs (Figure 4). A number of conserved sites among the ROPN1L orthologs extending up to ~150 residues beyond the docking domain must also be important for the correct protein functioning (Additional file 2). The insect ROPN1L orthologs are up to three times longer than the orthologs from other organisms due to a single highly variable insertion region. Its significance remains unknown, because apart from the N-terminal AKAP-binding domain, insect proteins do not contain any recognizable motifs.

Surprisingly, no proteins similar to AgROPN1L were identified in the *Drosophila melanogaster *genome. Similarly, no significant hits were returned from a TBLASTN search of the 12 *Drosophila *genomes available in the NCBI's WGS database. Although ROPN1L may have diverged in the *Drosophila *lineage past detection by BLAST search, this scenario seems unlikely taking into account conservation of the protein sequence in other animal groups. Alternatively, it could have been lost in the *Drosophila *ancestor, which implies that the presumed mechanism of sperm flagellum motility control in *Drosophila *may be different than in other insects. Remarkably, no sequences homologous to *AgRopn1l *were identified in *Caenorhabditis *genomes, but, unlike in *Drosophila*, sperm cells in nematodes are aflagellate, have amoeboid shape and move using pseudopods [40]. Moreover, *Caenorhabditis *lacks motile cilia [41], which, in other organisms, share elements of molecular motor machinery with flagella.

### AgDzip1l

The *AgDzip1l *is relatively well conserved. At the amino acid level, it shares 58% similarity with culicine sequences and 46% similarity with its human putative ortholog DAZ interacting protein 1-like protein (DZIP1L). DZIP1L remains uncharacterized and its function is unknown, but the Gene Ontology terms associated with its GenBank record include: "nucleic acid binding", "protein binding", "zinc ion binding", and "germ cell development". In addition, according to the UniGene EST Profile Viewer, the *Dzip1l *gene is expressed in a number of tissues, including testis. The DZIP1L shares 58% sequence similarity with the human DAZ interacting protein 1 (DZIP1), which is expressed in testis and co-localized with Deleted in Azoospermia (DAZ) protein playing a pivotal role in the male germ cell development [42].

The AgDZIP1L predicted protein contains a single C2H2 zinc finger domain. C2H2 zinc finger proteins comprise a large family of regulatory proteins involved in binding nucleic acids or proteins [43], and most of them contain between 2 and 37 C2H2 domains. [44]. In those that possess a single domain, high affinity DNA binding is achieved by a zinc finger complemented by short stretches of highly basic amino acids flanking the domain [45,46]. In AgDZIP1L, a highly basic sequence region found at the N-terminal flank of the domain (Additional file 2) is consistent with a putative AgDZIP1L involvement in nucleic acid binding, which is further supported by the WoLF PSORT analysis suggesting localization of the protein to the nucleus.

### Ams

*Ams *appears to be an extremely rapidly evolving gene in *Anopheles*. Because of its divergence, the TBLASTN search failed to identify its homologs even in other mosquitoes. Since such a search strategy may not be sensitive enough to detect highly diverged sequences, we used microsynteny information in an attempt to identify the *Ams *orthologs in culicines. *Tango1 *(*Transport and Golgi organization 1*) and *xdh *genes, which flank the *Ams *in *An. gambiae*, have conserved linkage in the culicinegenomes. The sequences spanning between *Tango1 *and *xdh *in *Aedes *and *Culex *share a short similarity region bearing a protein coding capacity, with ORFs in the same orientation as the *A. gambiae Ams *relative to the flanking genes. Using RT-PCR and species-specific primers encompassing the most conserved culicine ORFs we confirmed that this hypothetical gene is expressed (in a female-biased pattern) in both *Aedes *and *Culex *(Figure 3). Two transcripts, differing in size due to an intron splicing or retention, were strongly expressed in the *Ae. aegypti *females, while in males strong expression corresponded to a transcript with an intron spliced out. In *Cx. quinquefasciatus *a single transcript with an intron retained was expressed in a female-biased fashion. In both *Aedes *and *Culex *intron retention leads to a transcript that may encode a truncated, but functional, putative protein. It is also possible that intron retention is a result of a faulty splicing event, and such a transcript, with a premature termination codon (PTC), is destined to degradation via the nonsense-mediated mRNA decay (NMD) pathway [47]. However, the latter scenario seems implausible for *Culex*, in which the only identified transcript form contains a potential PTC. The culicine sequences are highly similar to each other at the amino acid level, but only remotely similar to the *Ams *gene (71%/90% identity/similarity, and 14%/31% identity/similarity, respectively; note, that the comparisons are based on a sequence fragment highly conserved in culicines, for which expression in *Aedes *and *Culex *could be experimentally confirmed; the complete culicine gene models were not created; see Additional file 2), leaving a large degree of uncertainty as to the homology between *Ams *and the putative culicine genes. However, because there is no other contradictory evidence, we regard these genes as putative orthologs.

The *An. gambiae *AMS protein is weakly basic (theoretical pI of 8.66). According to the TRESPASSER program analysis, it contains a leucine zipper motif (Leu-X_6_-Leu-X_6_-Leu-X_6_-Leu), which is found in α-helical segments. Leucine side chains from such segments can interact with other α-helices to form coiled coils. Existence of a coiled coil domain encompassing the leucine zipper motif is indeed predicted in the AMS protein by the COILS program from the PredictProtein package. Coiled coil proteins exhibit a wide range of functions, including cellular motility, signal transduction and modulation of transcription [48]. It should be noted, however, that because leucine is the most frequent amino acid and the motif is very simple, such a pattern may have arisen in the AMS protein by chance [49]. No leucine zipper motifs have been found in the culicine orthologs, but their large divergence from the *An. gambiae Ams *in both sequence and expression pattern implies that the proteins in culicines have attained different structure and function.

### mts

Sequences homologous to *mts *could be identified only in mosquitoes. The *mts *transcript encodes a protein that contains no identifiable motifs that would allow us to infer its function. However, the highly basic character of MTS (theoretical pI of 10.4) provides a hint about its potential involvement in chromatin condensation. Detection of *mts *transcription in spermatids (Figure 2C) further strengthens this supposition. In mammalian spermiogenesis a set of highly basic proteins is involved in chromatin restructuring, associated with a displacement of histones by transition proteins, which are subsequently replaced by protamines [50]. In effect, chromatin becomes transcriptionally silent and tightly packaged into a sperm nucleus, which is reduced in volume more than 20 times [51]. During maturation of the *Drosophila *sperm, the volume of the nucleus is reduced over 200-fold [52], apparently via similar mechanisms as those operating in mammals. Chromatin condensation in *Drosophila *is also associated with the removal of histones and accumulation of protamines. A temporal gap exists between the presence of histones and protamines and during that time a highly basic protein Tpl^94D^, presumably a functional homologue of mammalian transition proteins, is transiently expressed in the nuclei [53]. There are several other highly basic *Drosophila *proteins expressed in testes and likely to be involved in sperm maturation, which may functionally correspond to MTS, however, their specific functions remain unknown [53,54].

### AAms

The RT-PCR experiments showed that the *AAms *gene encodes two transcripts expressed in testis (Figure 1). One transcript consists of two exons coding for a 1188-residue protein. The second transcript, characterized by a shorter ORF generated by a transcript-specific intron splicing, may encode a truncated protein form (cf. Additional file 1). Alternatively, it may represent a misspliced transcript that bears a premature termination codon and, as such, is subject to degradation by NMD.

Sequences homologous to *AAms *were identified only in mosquitoes, in which they exhibit substantial variability in length and sequence (Table 1 and Additional file 2). The predicted AAMS protein orthologs lack any clearly defined conserved domains that would suggest their function or subcellular localization. The TopPred program predicted two transmembrane domains in the long version of the protein and one domain in its putative truncated form in *Anopheles*, two domains in its *Culex *ortholog, and between one and three domains in the *Aedes *protein, however, no such domains were identified in any of these proteins by the TMHMM program. The protein is acidic (theoretical pI of 4.94) and according to secondary structure and globularity prediction programs from PredictProtein server, half of its length forms loops interspersed among helices and strands, and its sequence folds into a compact structure.

## Conclusion

Our study sheds light on the virtually uncharacterized developmental processes in male mosquitoes. The strategy applied allowed us to identify five genes expressed in testes during late phases of spermatogenesis. The high complexity of testis function dictates that the number of genes involved must be much larger. In *D. melanogaster *there are over 1700 genes thought to be testis-specific [7]. Only about a 1000 of those have homologs detectable in *An. gambiae *(unpublished). Because the diversity of testis transcripts in mosquitoes is expected to be comparable to that in *Drosophila*, we predict that in addition to these conserved genes (it should be noted that some mosquito homologs may not share testis-specific expression patterns observed in *D. melanogaster*), many rapidly evolving genes, not detectable by a simple homology search, are expressed in mosquito testes. Further analysis of the SSH products using a more elaborate differential screening protocol is likely to provide more male-specifically expressed genes in *An. gambiae*. Likewise, further studies targeting transcripts from dissected testes are expected to give more information about testis-specific expression. Microarray analysis seems an attractive alternative to cDNA subtraction, however, the results in this method rely exclusively on the quality of microarray probes. It is likely that a considerable percentage of fast evolving genes expressed in *Anopheles *testes still awaits discovery (four out of five genes identified in the current study remain unannotated in the most recent *An. gambiae *genome release, which may be indicative of a problem). By definition, probes corresponding to such genes are absent from gene-based microarrays, therefore, their expression would remain undetected. This study demonstrates that the existing gene annotations can be further improved by the careful comparative analysis of the available mosquito genomes. Yet, because of a deep phylogenetic divergence reaching 145–200 million years [55], detection of rapidly evolving genes or correct delineation of their coding regions constitutes a major challenge in sequence comparisons between *Anopheles *and culicines. Consequently, further refinement of the *Anopheles gambiae *genome annotation may require genome sequences of additional *Anopheles *species and, still, supporting experimental evidence seems unavoidable.

The discovery of testis-expressed orphan genes opens new prospects for the development of innovative mosquito control methods. The proteins encoded by the orphans are unique in their potential as mosquito-specific targets of sterilizing chemicals. Such sterilizing insecticides could be a very useful alternative to irradiation in the production of sterile males for their subsequent release in the sterile insect technique approach to control. Furthermore, following proper testing, these chemosterilants could potentially be applied for direct control of wild mosquito populations and become an important component of an integrated management program for *An. gambiae*.

## Methods

### Insects

*Anopheles gambiae *G3 and Kisumu, *Aedes aegypti *Liverpool and *Culex quinquefasciatus *Thai laboratory strains were used in the experiments. Mosquitoes were reared according to established procedures [56-58].

### RNA isolation

RNA was extracted from all postembryonic developmental stages and selected male tissues of *Anopheles gambiae*, as well as from the *Culex quinquefasciatus *pupae and adults, and *Aedes aegypti *adults. *A. gambiae *adult males were dissected in 1 × PBS (Ambion). Heads, thoraces and abdomens (with undissected internal organs) were homogenized in TRI reagent (Sigma) and stored at -80°C until RNA extraction, whereas dissected midguts, Malpighian tubules, testes and accessory glands were stored in RNA *later *(Ambion) at -20°C. Prior to RNA extraction, larvae and pupae were washed twice in DEPC-treated water and larvae sexed by PCR using Y chromosome-specific primers [59]. Total RNA from developmental stages and body sections was extracted according to [60], with minor modifications. Following extraction, the samples were treated with TURBO DNase (Ambion) and the enzyme was removed by addition of DNase Inactivation Reagent (Ambion). The RNA solution was quantitated using ND-1000 spectrophotometer (NanoDrop Technologies), and stored at -80°C until needed. Total RNA from dissected tissues was extracted using RiboPure Kit (Ambion) according to manufacturer's specifications.

### cDNA subtraction

Total RNA was extracted from 100 whole female pupae and from distal segments of the abdomens (containing reproductive organs) of 200 male pupae using TRI reagent (Sigma) according to manufacturer's recommendations. An Oligotex mRNA Kit (Qiagen) was used to isolate poly A+ mRNA, from which cDNA was synthesized. Based on the assumption that the reproductive organ-specific transcripts may be relatively rare in the extracted mRNA population, the male cDNA was amplified using a SMART PCR cDNA Synthesis Kit (Clontech). Liquid hybridization was performed using 25 ng of the male and 1 μg of the female double stranded cDNA. Sequences expressed specifically/preferentially in males were enriched using a PCR-Select cDNA Subtraction Kit (Clontech) according to the manufacturer's protocols. The resulting cDNA subtraction products were run on an agarose gel. Detectable fragments were excised from the gel and reamplified by PCR using Nested Primer 1 and Nested Primer 2R included in the subtraction kit. The resulting PCR products were gel purified using QIAquick Gel Purification kit (Qiagen), ligated into pGEM-T Easy plasmid (Promega), and after electroporation, amplified in *E. coli *ElectroMAX DH10B cells (Invitrogen). Cloned templates were PCR amplified and sequenced using ABI BigDye terminator chemistry (PE Applied Biosystems) on an ABI 3130xl Genetic Analyzer.

### RT-PCR

The expression patterns of each subtraction product were tested in RT-PCR reactions using product-specific primers. SuperScript III One-Step RT-PCR System with Platinum Taq (Invitrogen) kit was used in all experiments. The reactions were performed in MyCycler thermal cycler (Bio-Rad) using 30 or 50 ng of total RNA and gene-specific primer pairs, with the following thermal conditions: 15 min of first strand cDNA synthesis at 50–60°C, followed by 2 min at 94°C, then 28–32 cycles of 94°C for 15 sec, 54–60°C for 30 sec, and 68°C for 40–120 sec, with the final extension at 68°C for 5 min. Fragments of the following mRNAs, with their respective primers in parentheses, were amplified to serve as an internal control of equal sample loading: the ribosomal *S7 *gene for *An. gambiae *(5'-TGCTGCAAACTTCGGCTAT-3' and 5'-CGCTATGGTGTTCGGTTCC-3') and *Culex quinquefasciatus *(5'-AGATGAACTCGGACCTGAAG-3' and 5'-TGCTGGTTCTTGTCCAGATG-3'), and *actin-1 *for *Ae. aegypti *(5'-CTGGAGAAGTCTTATGAACTTCCTGATGGTC-3' and 5'-GAATAGTATCTTCCGATGGGGATGTTCGTTAG-3').

### Amplification of cDNA ends

Amplification of 5'- and 3'- ends of the genes was performed with the GeneRacer kit (Invitrogen). Total RNA (2 μg) from abdomens of newly emerged adult males was dephosphorylated with calf intestinal phosphatase, followed by removal of the 5' mRNA cap structure using tobacco acid pyrophosphatase, which leaves a 5' phosphate only in full length mRNAs. Then, the GeneRacer RNA Oligo was ligated to the 5'ends of the mRNAs using T4 RNA ligase. The ligated mRNA was reverse transcribed using GeneRacer Oligo dT Primer and Superscript III reverse transcriptase. Thus obtained first strand cDNA was used to amplify the 5' end of each gene using a reverse gene-specific primer and the GeneRacer 5' Primer. PCR reactions contained 1 μl of the first strand cDNA reaction mixture with an initial denaturation at 94°C for 2 min, followed by 5 cycles of 94°C for 30 sec and 72°C for 3 min, then by 5 cycles of 94°C for 30 sec and 70°C for 3 min, then by 25 cycles of 94°C for 30 sec, 68°C for 30 sec, and 72°C for 3 min, followed by a final elongation step at 72°C for 10 min. The resulting products were subjected to a second round of PCR using the GeneRacer 5' Nested Primer and reverse gene-specific nested primers. Nested PCR contained 1 μl of the initial PCR reaction mixture as a template and was performed with an initial 94°C for 2 min, followed by 30 cycles of 94°C for 30 sec, 58–62°C for 30 sec, and 72°C for 3 min, with a final elongation step at 72°C for 10 min. The 3'-ends of the genes were recovered using gene-specific primers and the GeneRacer Oligo dT Primer, and the thermal conditions as given above for nested PCR (but the annealing temperature being 52–60°C). The PCR products containing both 5' and 3' ends were gel purified and cloned. Multiple clones from each PCR were sequenced as described above.

### Sequence analysis

Full length sequences of *An. gambiae *genes were assembled from multiple clones and verified by visual inspection of electropherograms using Sequencher v. 4.1 (Gene Codes Corp). Similarity searches against NCBI's and VectorBase databases were performed using BLAST programs [61]. Orthologs in *Ae. aegypti *and *Cx. quinquefasciatus *genomes were designated based on the reciprocal best hits in TBLASTN searches and on microsynteny (co-linearity and same orientation of flanking genes) information. Coding regions in culicines were identified using comparisons to the *An. gambiae *sequences and further validated using RT-PCR and sequencing of the amplified products; the validation was crucial for the correct delineation of the intron-exon boundaries. In more distantly related organisms no synteny evidence could be found and in such cases putative orthologs were identified based on the reciprocal best hit criterion. Homologs of *D. melanogaster *testis-specific genes were identified based on the best hits in TBLASTN searches of *An. gambiae *genome. The pattern discovery tool from the RSAT program [62] was used to search for 6–8 bp long putative testis-specific *cis*-regulatory motifs overrepresented in the upstream regions of the *An. gambiae *genes identified in this study. Multiple sequence alignments were performed using ClustalX [63]. Computation of the theoretical isoelectric point (pI) and the molecular weight was performed using the ExPaSy tool . Pfam , Prosite  and SMART  databases were searched to identify conserved domains in the predicted protein products. PredictProtein  was used for prediction of various aspects of protein structure and function. Searches for transmembrane helices were additionally conducted using Top-Pred  and TMHMM . SignalP  and WoLF PSORT  were used to characterize a potential for secretion and a likely subcellular localization of the proteins. Search for leucine zipper motifs was performed using TRESPASSER .

### Whole-mount in situ hybridization

A 354 bp fragment of *Ams *was amplified using primers cDNA3F (5'-TGGAACAGTTCAACAATGGG-3') and cDNA3R (5'-GGTCGATGATTTCCCGATTC-3'), and a 519 bp fragment of *mts *was amplified using primers cDNAsub4F (5'-ACCTTTCCGTTCCTCTTCAT-3') and cDNAsub4R (5'-ACTAAGCACAAGCAAAGCCC-3'). The PCR products were ligated into the vector plasmid pGEM-T easy (Promega) and the orientation of the cloned inserts was verified by sequencing. The plasmids were linearized by complete digestion with either *Spe*I or *Nco*I to generate templates for sense and antisense probes. Linearized plasmids were used for *in vitro *transcription with T7 or SP6 RNA polymerase to create sense and antisense DIG-labeled probes according to [64]. Testes and accessory glands of adult males < 12 h posteclosion were dissected in PBS and processed for hybridization following [64]. Probe was detected in a color reaction induced by alkaline phosphatase conjugated to anti-DIG antibody. The activity of endogenous alkaline phosphatases was not eliminated prior to probe detection, which resulted in the induction of a low level of background color reaction. After signal detection samples were washed for 5 min in PBT (PBS, 0.1% Tween 20) to stop the staining reaction, then stored overnight in 70% glycerol and mounted the next day on slides.

## Authors' contributions

JK designed the study. Both authors performed the experiments, analyzed the data and wrote the manuscript. Both authors read and approved the manuscript.

## Supplementary Material

Additional file 1 **Genomic context and EST evidence for the identified genes, primer sequences used for the RT-PCR analyses of their expression in *An. gambiae*, *Ae. aegypti *and *Cx. quinqufasciatus*, and details on SSH fragments lacking male-biased expression.** Click here for file

Additional file 2 **Amino acid sequence alignments.** Click here for file
