# Supplementary material for: Analysis of expression in the Anopheles gambiae developing testes reveals rapidly evolving lineage-specific genes in mosquitoes
Source: BMC Genomics. 2009 Jul 6;10:300. doi: 10.1186/1471-2164-10-300 (PMC2713267; doi:10.1186/1471-2164-10-300)
Supplement: Additional file 1 — Genomic context and EST evidence for the identified genes, primer sequences used for the RT-PCR analyses of their expression in An. gambiae, Ae. aegypti and Cx. quinqufasciatus, and details on SSH fragments lacking male-biased expression. [file 1471-2164-10-300-S1.doc]

**Additional file 1**

**Genomic context and EST evidence for the identified genes**

***AgROPN1L***

The *AgRopn1l* gene, located on chromosome 2R (chromosomal division 19C), is composed of 7 exons containing a 2040 bp open reading frame (ORF) that encodes a 679 amino acid protein. Its 3’UTR lacks a standard polyadenylation signal, but a potential alternative hexamer (AATACA) is found 28 bp upstream from the polyA tail.

A BLASTN search of the NCBI EST database yielded two hits matching the *Ropn1l* ortholog from *Ae. aegypti*. The ESTs were derived from adult testes and adult females, consistent with the results of our RT-PCR experiments. In addition, a 160 bp long EST (GenBank accession no. BM635560) derived from *An. gambiae* pooled mixed-sex whole adults was identified to partly match the 3’UTR of the *AgRopn1l* cDNA. However, this EST is apparently a chimeric artifact of the library construction, because it could not be aligned in its entirety with sequences from any single *An. gambiae* chromosome.

***AgDzip1l***

The *AgDzip1l* gene located on chromosome 2R (7A) consists of two exons harboring a 2544 bp ORF, which encodes an 848 amino acid protein. It corresponds in part to a predicted gene AGAP001165, which is truncated relative to the *AgDzip1l* ORF and terminates 723 bp upstream from the actual stop codon. The *AgDzip1l* ortholog from the *Culex* genome (VectorBase accession no. CPIJ011569) has also been incorrectly predicted at the 3’ end, as judged from its comparisons to the *Anopheles* and *Aedes* sequences identified in the present study (Additional file 2; Supplementary Fig. 2). Within the *An. gambiae* genome context, the *AgDzip1l* overlaps at both ends the untranslated regions of the flanking genes (AGAP001166 encoded on the same strand as *AgDzip1l* and AGAP001164 encoded on the reverse strand; the *AgDzip1l* overlapping regions of both genes have supporting EST evidence). The *AgDzip1l* transcript contains a standard polyadenylation signal 30 bp upstream from the polyA tail.

No *An. gambiae* ESTs corresponding to the *AgDzip1l* have been deposited in the NCBI EST database prior to this study. Five ESTs derived from *Ae. aegypti* first instar larvae and adult females were identified to match the *AgDzip1l* ortholog, which accords with a relatively high sex-unbiased expression observed in *Ae. aegypti* adults and is consistent with the expression of the gene in all *An. gambiae* life stages.

***Ams***

The *Ams* gene is located on chromosome 3R (29C) and is flanked from the 5’ end by the *Tango1* (*Transport and Golgi organization 1*) gene (at a distance of 202 bp) and from the 3’ end by the *xdh* gene (at a distance of 31 bp). It consists of 3 exons and contains a 1227 bp ORF encoding a 408 amino acid protein. The transcript contains an AATATA hexamer 27 bp upstream from the polyA tail, which may serve as the polyadenylation signal.

Three *An. gambiae* ESTs deposited at NCBI match fragments of the *Ams* cDNA sequence, but their analysis shows that only one EST derived from whole unsexed adults corresponds to an *Ams* transcript. Two other ESTs represent fragments of the overlapping 3’UTR of a downstream *xanthine dehydrogenase* (*xdh*) gene encoded on the reverse strand and whose coding sequence ends only 31 bp from the end of the *Ams* transcript. Despite the EST evidence, the gene has not yet been annotated and included in the *An. gambiae* gene build. No ESTs corresponding to *Ams* ortholog from *Aedes* have been found at NCBI.

***mts***

The *mts* gene consists of two exons and contains an 897 bp ORF encoding a 295 amino acid protein. A BLASTN search of the *An. gambiae* PEST strain genome using the full length cDNA as a query resulted in two hits. One hit corresponds to a scaffold (AAAB02008898) mapped to the chromosome 2R (19C). The other hit is to a short unmapped scaffold (AAAB01000967) that evidently represents an alternative assembly of the same genomic region, because Southern blot analysis does not support the presence of two gene copies within the genome (data not shown). Interestingly, two copies of the homologous gene were found in the *Aedes* genome (within genomic supercontigs 1.453 and 1.414). High nucleotide sequence identity (96%) of both copies within both coding and non-coding regions suggests that the duplication in *Aedes* may have occurred very recently.

The *An. gambiae mts* transcripts were not represented in the NCBI EST database at the time of our study, however, three *Ae. aegypti* ESTs derived from testis and eight from the females infected with *Bruggia malayi* and dengue virus were identified to match the *mts* ortholog. The EST data from females contradict our RT-PCR results, which indicate male-specific expression of that gene in *Aedes*. It is conceivable that transcripts detected in *Aedes* females result from misexpression of the gene due to infection, although currently this supposition remains speculative.

***AAms***

The *AAms* gene is located on the chromosome 2R (16E). According to the RT-PCR results, the gene encodes two transcripts expressed in testis (Fig. 1). One transcript consists of two exons, which harbor a 3567 bp ORF coding for a 1188-residue protein. The second transcript, characterized by a shorter ORF generated by a transcript-specific intron splicing event (cf. Additional file 2; Supplementary Fig. 5), apparently encodes a truncated protein form (since the structure of this transcript was not analyzed in details, we do not have any experimental evidence regarding its ends; however, the protein truncation is suggested by the in-frame stop codon present 41 nucleotides downstream from the splice acceptor site).

No ESTs corresponding to the *AAms* gene or its orthologs were submitted to the NCBI database prior to our study.

**Table 1. Mosquito ESTs deposited at NCBI and searched using full length sequences of genes identified in this study as queries.**

|  | cDNA source | | | | |
| --- | --- | --- | --- | --- | --- |
|  | Testis | Unsexed preimaginal stages | Mixed sex whole adults | Whole adult females and female tissues | Total |
| *An. gambiae* | - | 23,686 | 82,926 | 46,553 | 153,165 |
| *Ae. aegypti* | 2,779 | 62,064 | - | 236,499 | 301,342 |
| *Cx. quinquefasciatus* | - | - | 57,262 | 147,480 | 204,742 |

**Table 2. Primers used for the RT-PCR analyses of expression of genes identified in this study (cf. Fig 1 and 2).** For each gene and species the forward (F) and the reverse (R) primer sequences are given in 5’-3’ orientation.

| Gene |  | *An. gambiae* | *Ae. aegypti* | *Cx. quinquefasciatus* |
| --- | --- | --- | --- | --- |
| *Ropn1l* | F  R | GCGTAGAAGAAAAACACAAAAGCAT  TGGCTGCGTTCGTATTACCG | GTTCGTCGATGGGAGAAAAG  TCACACTCCCCGTCGATAAT | TGTGGATGTTCAAGGAGTGC  AAATCGCCACCGTACTCAAC |
| *Dzip1l* | F  R | GGCCAAAGTGATACAAATTGTTT  CGTTTCCAATAGGGACTTCG | CGAATGAAGCCAAACAAGACC  AGAGACATTACTTCGTGACAGC | AGTTTCGCCAAAGAAGCAGA  GTTGTGCCGCTACCTTGTTT |
| *Ams* | F  R | CATACGGGAGGTGAGGAAAT  CCCCTTCATGCTTCATCTT | TTCGAGACGCTCAAGTACGA  CTCACGGTCCTTTTCGATGT | TTCGAGAGTCTCAAGCACGA  CCAGCTCGTAGTCCTTTTCG |
| *mts* | F  R | TGGGATCCAAATTATTTCGTG  CTGTTCGGTTCAACAATGGA | CGCTAATTCCGGAGTGAAAA  GGGGATCGATTTACCCAGAT | GAGAATTCCTCCGTGACAGC  ATGGCACCATCAGTTTCCTC |
| *AAms* | F  R | ACCCTACACCTGCTTCTTCG  CGCACTCCATCACCGATTC | CGATCCTCCGGAGTTAACAA  TGTGCAACGACTCTTGAAGG | TGAAGCACAGCCTATCGTTG  CGTTTGCTGGAAAAGTACCC |

**Table 3. Details on SSH fragments lacking male expression bias.**

| Clone | GenBank accession | Annotation status^1^ | Female EST | Best Blastx match | % identity/E value | Homologue of known function |
| --- | --- | --- | --- | --- | --- | --- |
| subC1 | GO479231 | **XM_563568.1** | No | AGAP002593 | 100/9e-45 | apolipoprotein |
| subC2 | GO479232 | [**XM_316348.4**](http://www.ncbi.nlm.nih.gov/entrez/query.fcgi?cmd=Retrieve&db=Nucleotide&list_uids=158295675&dopt=GenBank&RID=Y77CBSHW016&log$=nucltop&blast_rank=1) | No | AGAP006283 | 100/6e-17 | cuticular protein 70 |
| subC3 | GO479233 | [**XM_313971.4**](http://www.ncbi.nlm.nih.gov/entrez/query.fcgi?cmd=Retrieve&db=Nucleotide&list_uids=158292552&dopt=GenBank&RID=Y77FRXA7011&log$=nucltop&blast_rank=1) | Yes | AGAP005095 | 100/8e-45 | beta-actin |
| subC4 | GO479234 | [**XM_001238121.2**](http://www.ncbi.nlm.nih.gov/entrez/query.fcgi?cmd=Retrieve&db=Nucleotide&list_uids=158299155&dopt=GenBank&RID=Y77ND23T013&log$=nucltop&blast_rank=1) | No | AGAP012875 | 95/ 3e-33 | cuticular protein 99 |
| subC7 | GO479235 | [**XM_319271.3**](http://www.ncbi.nlm.nih.gov/entrez/query.fcgi?cmd=Retrieve&db=Nucleotide&list_uids=119114454&dopt=GenBank&RID=Y77VD22Z016&log$=nucltop&blast_rank=1) | No | AGAP010117 | 96/3e-40 | cuticular protein 95 |
| subC9 | GO479236 | [**XM_315091.3**](http://www.ncbi.nlm.nih.gov/entrez/query.fcgi?cmd=Retrieve&db=Nucleotide&list_uids=118786038&dopt=GenBank&RID=Y7826BKS011&log$=nucltop&blast_rank=1) | Yes | AGAP004987 | 98/ 2e-50 | - |
| subC10 | GO479237 | [*XM_001238567.2*](http://www.ncbi.nlm.nih.gov/entrez/query.fcgi?cmd=Retrieve&db=Nucleotide&list_uids=158302428&dopt=GenBank&RID=Y785RS5T011&log$=nucltop&blast_rank=1) | Yes | AGAP001174 | 99/6e-72 | 14.5 kDa salivary peptide |
| subC11 | GO479238 | [*XM_312231.3*](http://www.ncbi.nlm.nih.gov/entrez/query.fcgi?cmd=Retrieve&db=Nucleotide&list_uids=118782387&dopt=GenBank&RID=Y788SFRB016&log$=nucltop&blast_rank=1) | No | AGAP002691 | 100/1e-17 |  |
| subC12 | GO479239 | [*XM_311486.4*](http://www.ncbi.nlm.nih.gov/entrez/query.fcgi?cmd=Retrieve&db=Nucleotide&list_uids=158289853&dopt=GenBank&RID=Y78E3MY801R&log$=nucltop&blast_rank=1) | Yes | AGAP010461 | 100/ 2e-15 | Histone H1 |
| subC13 | GO479240 | [*XM_312551.2*](http://www.ncbi.nlm.nih.gov/entrez/query.fcgi?cmd=Retrieve&db=Nucleotide&list_uids=58383449&dopt=GenBank&RID=Y78JZB3W011&log$=nucltop&blast_rank=1) | Yes | AGAP002401 | 100/3e-32 | vacuolar ATP synthase subunit e |
| subC15 | GO479242 | Unannotated | Yes | - | - | - |
| subC16 | GO479243 | Unannotated | Yes | - | - | - |
| subC17 | GO479244 | [*XM_313417.4*](http://www.ncbi.nlm.nih.gov/entrez/query.fcgi?cmd=Retrieve&db=Nucleotide&list_uids=158291889&dopt=GenBank&RID=Y78YDCBT01R&log$=nucltop&blast_rank=1) | Yes | AGAP003649 | 90/4e-30 | zinc finger protein |
| subC18 | GO479245 | [**XM_312474.4**](http://www.ncbi.nlm.nih.gov/entrez/query.fcgi?cmd=Retrieve&db=Nucleotide&list_uids=158290946&dopt=GenBank&RID=Y791HXTN01R&log$=nucltop&blast_rank=1) | Yes | AGAP002465 | 98/6e-51 | ferritin heavy chain-like protein precursor |
| subC19 | GO479246 | [**XM_318947.4**](http://www.ncbi.nlm.nih.gov/entrez/query.fcgi?cmd=Retrieve&db=Nucleotide&list_uids=158298781&dopt=GenBank&RID=Y797WCS601R&log$=nucltop&blast_rank=1) | Yes | AGAP009833 | 98/2e-39 | voltage-dependent anion-selective channel |
| subC20 | GO479247 | [**XM_314556.3**](http://www.ncbi.nlm.nih.gov/entrez/query.fcgi?cmd=Retrieve&db=Nucleotide&list_uids=118785340&dopt=GenBank&RID=Y799W4B0016&log$=nucltop&blast_rank=1) | Yes | AGAP010591 | 99/4e-61 | 40S ribosomal protein S20 |
| subC21 | GO479248 | [*XM_320350.4*](http://www.ncbi.nlm.nih.gov/entrez/query.fcgi?cmd=Retrieve&db=Nucleotide&list_uids=158300424&dopt=GenBank&RID=Y79C1H34011&log$=nucltop&blast_rank=1) | Yes | AGAP012185 | 98/1e-66 | coracle protein |
| subC22 | GO479249 | [**XM_559853.2**](http://www.ncbi.nlm.nih.gov/entrez/query.fcgi?cmd=Retrieve&db=Nucleotide&list_uids=118780264&dopt=GenBank&RID=Y79EAT13011&log$=nucltop&blast_rank=1) | Yes | AGAP009368 | 100/ 6e-17 | - |

^1^GenBank accession numbers of annotated mRNAs that match full length of the SSH fragments are given in bold, those that match portions of the SSH fragments are given in italic.
