# Supplementary material for: Analysis of expression in the Anopheles gambiae developing testes reveals rapidly evolving lineage-specific genes in mosquitoes
Source: BMC Genomics. 2009 Jul 6;10:300. doi: 10.1186/1471-2164-10-300 (PMC2713267; doi:10.1186/1471-2164-10-300)
Supplement: Additional file 2 — Amino acid sequence alignments. [file 1471-2164-10-300-S2.doc]

**Additional file 2**

**Amino acid sequence alignments**

For each alignment, amino acids identical in at least half of the taxa in a given alignment position are shaded black and those with similar physicochemical properties are shaded grey. The organism names were abbreviated as follows: *Hs* – *Homo sapiens*, *X. laevis* – *Xenopus laevis* (frog), *O. mossambicus* – *Oreochromis mossambicus* (fish), *S. purpuratus* – *Strongylocentrotus purpuratus* (sea urchin), *T. castaneum* – *Tribolium castaneum* (beetle), *P. humanus* – *Pediculus humanus* (louse), *Ae. aegypti* – *Aedes aegypti*, *Cx. quinquef.* – *Culex quinquefasciatus*.

**Supplementary Fig. 1**. Amino acid sequence alignment of the selected putative orthologs of *AgRopn1l*. Positions conserved across all the taxa are marked with asterisks, and the ones conserved in mosquitoes are additionally marked with the caret (^) sign.

*Hs_*ROPN1L MP-LPD--TMFCAQQIHIPPELPDILKQFTKAAIRTQPADVLRWSAGYFSALSRGDPLPV
*X. laevis*  MP-PPE--TMFCAQQINIPPELPDILKQFTKAAIRTQPHDLLQWSAAYFDSLSKGEPLPV
*O. mossambicus*  MP-LPD--TFYCAQQINIPPELPDILKDFTKAAIRTQPKDLLQWSVAYFSALSKGECLPV
*S. purpuratus*  MPGIGDDVPMYCSQQINIPPDLPDIMKQFTKSAIRTQPADVLQWSAAYFHALANGETPPV
*T. castaneum*  MPEMGA--DMYCSQQICIPPTFPYLLRQFAKAAIRTQPNDLLKWATTYFRCLSLNIPPPV
*P. humanus*  MPDLLD--RIYCSEQIHIPPTFPYIMKLYCKAAIRTQPYDLLKWSAAYFRALANGEEPPV
*Ae. aegypti*  MVLLSE---IYCPEQIVIPENFNNVLKVYAKAVIRTQPFDLLRWSAAYFRCLDLEKAPPV
*Cx. quinquef.* MVLLPE---IYCPEQIVIPENFNNVLKVYAKAVIRTQPFDLLRWSAAYFRCLALERTPPV
AgROPN1L MVLLQE---IYCPEQIVIPENFHITLKQYAKAVIRTQPFDLLRWSAAYFRSLALSVQPPV

*^^^ ^ ^^*^^**^**^^^ ^^ ^^*^^*****^*^*^*^^^**^ * ^ ^**

*Hs_*ROPN1L KDRMEMPTATQKTDTGLTQGLLKVLHKQCHHKRYVELTDLEQKWKNLCLPKEKFKALLQL
*X. laevis*  KDRVELQVATQKTDSGLTPGLLKVLNKQLSSKMSVKIADLKQKWTDLCLPEEQLQNILGL
*O. mossambicus*  XDRLELNVATQKTDTGLTPGLLKILYKQLSPKKTCSKEELXAKWKGQCLPVAQLETLLSL
*S. purpuratus*  KERLEMPMATQKTDTGLTPGILSVLNRQLGPKKTISVDELEQKWKDNCLPKERMFSLIQI
*T. castaneum*  KPRLEYPIP--RDHHGLTPGWLRALLYQMAGHQTVTFKTLWDRWIGACLQHPNLIQILVL
*P. humanus*  KERIEFPPY--DSPSGLTPGYIKMLINQVS----YRAQTLFKKWSDVSLQEMLLIKLIAL
*Ae. aegypti*  KPRYE--PEPSRGR--LSTGALRVLIDQLGKGYFVQKRILQDKWQGLCLPEDDLLNILSL
*Cx. quinquef.* KPRYE--PELRRGR--LSGGALRVLIDQLGKGFFVQKRILQEKWQGLCLPEDDLLNLLSL
AgROPN1L KSRYE--PAGHFRQ--LTIGIVRVLIDQLGKGYYVHKKILLEKWEGLCLPEEDLLNVLSL
 ^ *^* ^ ^ * ^^*^^^^^^^ ^ ^^ ^* ^^^*^^ ^^^^ ^^^

*Hs_*ROPN1L DP--CENKIKWINFLALGCSMLGGSLNTALKHLCEILTDDPEGGPARIPFKTFSYVYRYL
*X. laevis*  DN--FQDDIDWLKFLSLGCSALGGSISSALKYACEILTEDPEGGAAHIPFDTFTYIYKYL
*O. mossambicus*  GS--FGSDIDWMEFFALGCSALGGTLISSLKFACEILTEDETGGAARIPFDTFVRLYTYL
*S. purpuratus*  GS--FGDEIDWRKFFALACSALAGNITSAMKVICEILTKDPEGGAARIPFDLFKELYTYL
*T. castaneum*  GGFDDNQAVPWLRFIALCAAHLTDSLTQTMILICEILTEEPEGGSAMIPLSTFLDLYEFL
*P. humanus*  LG--AVTSINWVQFVGVCAGFISNTLSQTMILICELFTEEPEGGMATIPF-----LKPFL
*Ae. aegypti*  LRMLDWSHLHWLKIVAVFIGLLSDSLPRTAEMICELLTEEPEGGPAPIPLWMFKECFLAV
*Cx. quinquef.* LRMLDWPQLHWLKIVAVFIGQLSDSLPRTAEMICELLTEEPEGGPAPIPLWMFKECFLAV
AgROPN1L LRMLNWSQLHWLKIIAVFIGQLCKNFTKTTEMICEMLTEDPDGGAASIPFWMFRECFLAL

^^^^ ^ ^^^^^^ ^^^^^ ^ ^ ^^^** ^*^ ^ ** * ** ^^ ^^^^^

*Hs_*ROPN1L ARLDS-------------------------------------------------------
*X. laevis*  AHIDG-------------------------------------------------------
*O. mossambicus*  AQLDG-------------------------------------------------------
*S. purpuratus*  AQIDG-------------------------------------------------------
*T. castaneum*  ARIDASLPQTLKNYYFLDKFLELFREKIAKDSGEKPVEEELETDFMSEIVSEVTEKSERE
*P. humanus*  CCLHC-------------------RKKIVLEDMEESIRAKIEAKLAEDEESTVDEQ----
*Ae. aegypti*  ARLDCGSVQTFVDGRKVLDG--GQLEPVRPMTELPKVLSTISFKNAIIDKYKNIMVNVQS
*Cx. quinquef.* ARLDCGALQTFVDGRKVLED--GQLERKRSMTEMPKVLSSVSFKNAIIDKYKSIMG-IDT
AgROPN1L ADLDCSSVQKFVNGRKVLDEDTGALEEEQMPPALPKVLSTISFKNAIIDKYRSTLEHETK
 ^ ^^^ ^ ^^ ^^^^^ ^ ^^ ^^^^^ ^^^^^^^^^^

*Hs_*ROPN1L ------------------------------------------------------------
*X. laevis*  ------------------------------------------------------------
*O. mossambicus*  ------------------------------------------------------------
*S. purpuratus*  ------------------------------------------------------------
*T. castaneum*  TKKDDEGSVVSCPSV---------------------------------------------
*P. humanus*  ------------------------------------------------------------
*Ae. aegypti*  STTFTDGELLFTEDR--FSEKSDVPSHLDSDFKFVGDETDPYEVVRRAPDFDSVIVLLGE
*Cx. quinquef.* KLDRGDAELLSVEER--FEELSEVPSRIDSDFRFVGDETDPVEVVRRAPDFESVIVLLDE
AgROPN1L QHHFALSGNINTDDNDQLSEPSVAPSRLESDFKFFGDDTDAHESLRRAPDFNSVIVLLKR
 ^ ^ ^^ ^^^ ^ ^^ ^^ ^ ^^^^^ ^^^^^^

*Hs_*ROPN1L ------------------------------------------------------------
*X. laevis*  ------------------------------------------------------------
*O. mossambicus*  ------------------------------------------------------------
*S. purpuratus*  ------------------------------------------------------------
*T. castaneum*  ------------------------------------------------------------
*P. humanus*  ------------------------------------------------------------
*Ae. aegypti*  LHDDKLKQQLSSVSISEEKLERAKERTQRLEDLKESLPEYDFEKLMEAERLQLLKDMGPP
*Cx. quinquef.* LREERLKEQLSTVAISEAKLERAKERSQRLEDLREGLPEYDYEKLMEAERQQLLKDMGPP
AgROPN1L LRGDGAISMVSESTLSKAALERTRERSKQIEELKVSISEEEYNKLKEQERHELLKEMGPP
 ^ ^ ^ ^^^ ^^ ^ ^ ^ ^^ ^ ^^ ^^^ ^^^^

*Hs_*ROPN1L ------------------------------------------------------------
*X. laevis*  ------------------------------------------------------------
*O. mossambicus*  ------------------------------------------------------------
*S. purpuratus*  ------------------------------------------------------------
*T. castaneum*  ------------------------------------------------------------
*P. humanus*  ------------------------------------------------------------
*Ae. aegypti*  WLLLYLFSKTTSRDRSFNYIEETSLEDEESIAGYSNDSYELRGAHKLDRSPEPEMSDEED
*Cx. quinquef.* WLLLYLFSRSTGRDRSYNYLEETSLEDEGSVAGFSNDSFDMRGAERLD--------VEEG
AgROPN1L WLLLXLXSQDCSRSKSFNYVEQSST----SISDDSVSAYSMENIFEDT---------LEL
 ^^^^ ^ ^ ^ ^^ ^ ^ ^ ^

*Hs_*ROPN1L ------------------------------------------------------------
*X. laevis*  ------------------------------------------------------------
*O. mossambicus*  ------------------------------------------------------------
*S. purpuratus*  ------------------------------------------------------------
*T. castaneum*  -----------------------------------TSQDPDNYMKLLMKIRAEGEFEESN
*P. humanus*  ----------------------------------------------------EQEFEELS
*Ae. aegypti*  DTEIKSSLGRRRSSYSIPASVRSRMSSVYSSSTRIANEVLSRIICMVDEAIIDGECELSV
*Cx. quinquef.* DEEYHNVERRRSSTYSMPASVRSRMSSIYSSATRIANEVLSRIICMVDEAIIDGECTLSV
AgROPN1L DPSATFAHDRRKSSFSLPVTLRARMSVSSHSAAFITNEILSKVVCSIDQAIIDGECELSL
 ^ ^^ ^ ^ ^ ^ ^^^ ^ ^ ^^ ^^ ^ ^ ^^^^^^^ ^

*Hs_*ROPN1L ------------------------------------------------------------
*X. laevis*  ------------------------------------------------------------
*O. mossambicus*  ------------------------------------------------------------
*S. purpuratus*  ------------------------------------------------------------
*T. castaneum*  EEIKEETEEKTEPVDEEVKEEEVEERKESVAVSEKKFPEQLAEDEKIPQFPVEDDLVSKS
*P. humanus*  SYLLGAAARVRKYTAEVLG--------------YKIFKKYMRQD----------------
*Ae. aegypti*  KSITSFVEQKSREDTN-ISPQDFGTLRDFLVEAEKKDLQVKDLNELYHFFVGESFKATGE
*Cx. quinquef.* ASIASFIERKSHDESARISEHDFSTLKDFMEEAQRKELEVKDLNQLYHFFVGESFKAAAD
AgROPN1L GSIANFLEEKSNSNEDFLAPADHEKLQSFLKEAESRELEVKDINELYNFFVSESFKRALQ
 ^^ ^ ^ ^^ ^ ^ ^ ^ ^^ ^ ^^^ ^ ^^ ^^^ ^^^^

*Hs_*ROPN1L ------------------------------------------------------------
*X. laevis*  ------------------------------------------------------------
*O. mossambicus*  ------------------------------------------------------------
*S. purpuratus*  ------------------------------------------------------------
*T. castaneum*  ------------------------------------------------------------
*P. humanus*  ------------------------------------------------------------
*Ae. aegypti*  NVDDQKRNRSREITSIFEASGIEVDNTLLDAHISENMILGLVHKDPEDHKEQVPSNPTVE
*Cx. quinquef.* ---EPKRDRSKEITSIFEASGIEVDNTLLDAQISENMILGLVHKESE----PVPT-PDLS
AgROPN1L--DNDNEPQQKEIFSIFGESGIEVDANLLEAKISDSMILGLVA----------P--PPIE
 ^^ ^^^ ^^^^^ ^^ ^^ ^^^^^^

*Hs_*ROPN1L ------------------------------------------------------------
*X. laevis*  ------------------------------------------------------------
*O. mossambicus*  ------------------------------------------------------------
*S. purpuratus*  ------------------------------------------------------------
*T. castaneum*  ------------------------------------------------------------
*P. humanus*  ------------------------------------------------------------
*Ae. aegypti*  VTDDEPKDEEAEEGASALEQELHTLVEVVEEAEPEQEAEPAPDVATQTDPLNVKCMEMMR
*Cx. quinquef.* PRQIPADEAVLHVTAPDLEAVPEDEEEPPEEHSEEQVSSP-PEVATQTDPLNVKCTELMR
AgROPN1L EPSEETKEKSESVELSAILPTPSMDTLLPIACEG----------ITQTDPIEIACVQ--R
 ^^^^^ ^ ^

*Hs_*ROPN1L --------------------------DVSPLETESYLASLKENIDARKNGMIGLSDFFFP
*X. laevis*  --------------------------DISEMQIEDVLNVLQSEAE-RQNGLIQPRNFLSS
*O. mossambicus*  --------------------------DVPQERMDNLPSSLQPQVN-KPNGMN--SGFHL-
*S. purpuratus*  --------------------------EISEEQIEGVFTYLQYHVD-KQNGAVQPRNFLHP
*T. castaneum*  -------TRMSSAPSESLKKVPGIGPKVPENLILAVCNYMKDVAK-WQHDMVMPRNIRHF
*P. humanus*  --------------------VPGIGPAIRSEQVEKVVDFMDFWSH-KQEEMVMPRNIRHW
*Ae. aegypti*  RRSLTRSADVMVEFSCRLPALPGIGPPLDEGTIVAFLAYLTKRAV-HQHGMIYPRNFREP
*Cx. quinquef.* RRTLTRSSELMVEYSCRIPPIPGIGPPLSEDVIQAFLGYLAKRAI-HQQGLIYPRNFRET
AgROPN1L KIPLVPTDDQRITYKCKISPIPGIGTPLDETVRDAFLTYLAQRAV-DQQGLIYPRNFQEA
 ^ ^ ^^^^ ^^ ^ ^^^ ^^ ^ ^ ^ ^^^^^^

*Hs_*ROPN1L KRKLLESIENSEDVGH
*X. laevis*  QCPLLS----------
*O. mossambicus*  ----------------
*S. purpuratus*  DCPKLGLM--------
*T. castaneum*  DRPPLEIRNQ------
*P. humanus*  MCPPLDKIPNPEP---
*Ae. aegypti*  PCPKLDSGEGEGK---
*Cx. quinquef.* PCPKIG----------
AgROPN1L LCPRIE----------
 ^^

**Supplementary Fig. 2.** Alignment of the human DZIP1L protein with orthologs from selected insect species. The C2H2 zinc finger domain is marked with red font color; a stretch of basic amino acids presumably required for the nucleic acid binding flanks the domain from the N-terminus and is marked in green. In *Tribolium* and *Drosophila* basic amino acid stretches are shorter, but several additional longer stretches are present at the carboxy terminus (not marked).

*Hs*_DZIP1L MQSPAATAEGLSGPLFGAYTFPTFKFQPRHDSMDWRRISTLDVDRVARELDVATLQENIA
*T. castaneum*  -MYKGDCKWHHDYVRLAFDTGFKFEKHKSDN-FDRNKISLIDIDRVIEERDFATVETLIP
*D. melanogaster*  ------MGFKGKYPQMVRETGFKLRQYRDGP-LDWRLMGSYETERILREQNFELVDKALT
*Ae. aegypti*  ----MAHKWNHNFPKIAREAGFVIKELSVGHCIDWRFIASIDPYAIVGDKDYEKLDEFIP
*Cx. quinquef.*  ----MSHKWNHNFPKIAREAGFAIRELSVGHCIDWRFIASIDPYSIVSERDYEKLDEFIP
*An. gambiae*  ----MSYKWHHNFPKIAREAGFTIRELSVGHCIDWRFIASIDPYGIVGDKDYEKLDEFIP

*Hs_*DZIP1L GITFCNLDREVCSRCGQPVDPALLKVLRLAQLIIEYLLHCQDCLSASVAQLEARLQTSLG
*T. castaneum*  GITKFVLDPDLA----QVLDVSFVKTFKLSQLAIQYLLFCKKYLDNTVIVLKDELKKVRG
*D. melanogaster*  HLSEAPLGTVLET---HILDSGIAKYFVMSQYAIQYLMCCRTYLDECVTDLKEAHTTAQE
*Ae. aegypti*  HIAEVPIGTVLNN---RILDPAIGKYFILAQFSIQYLLFCKRFLDETVVEIRNTAQDVQK
*Cx. quinquef.*  HISEVPIGSVLNN---RILDPAIGKYFVLAQFSVQYLLFCKQFLDETVMEIRNTAQDVQK
*An. gambiae*  HISEVPIGNVLSN---RILDPAIGKYFVLAQFSIQYLLFCKQFLDETVLEIRNTIQTLQD


*Hs_*DZIP1L QQQRGQQELGRQADELKGVREESRRRRKMISTLQQLLMQTGTHSYHTCHLCDKTFMNATF
*T. castaneum*  ENKELNLFVDDLKEHIATLMRE-------IDEKVA----------FKCEQCLKVFSSEEF
*D. melanogaster*  EIATLRKSLSESNNEVVQLHKR-------ITQIEAIREVV-----YPCHLCTKNFISNEA
*Ae. aegypti*  ENSRLEKIRRKKNEEIVSLHRK-------LQRAESAHAQQ---LVFPCSKCTKNFISLEL
*Cx. quinquef.*  ENARLEKICRKKNDELMTLHRR-------LQRAENVQIQQH--AVYPCSKCTKNFISSEL
*An. gambiae*  ENARLEKMNKKRSEEVTQLQRK-------LQRAETMEHHQHAQQIYPCTKCTKNFISSEL


*Hs_*DZIP1L LRGHIQRRHAG------------------VAEGGKQKKQEQPVEEVLEELRAKLKWTQGE
*T. castaneum*  LNSHIRRRHS----------------------TPTQVPETDKLQMEIKELKGRLNDAEKM
*D. melanogaster*  LNVHIGRKHRVASPPSLTSATG--KEKDRDKATDVHLINTIKMELEIKQLKERLNAAERN
*Ae. aegypti*  LNAHMARKHAEMGTNVATSGEP-KPAGRKLSETDSNLINTIKLELEVKQLKERLNATEKD
*Cx. quinquef.*  LHSHMVRKHG-----VSHEPEPSVTAGRKLSETDSNLINTIKLELEVKQLKERLNVAEKD
*An. gambiae*  LNAHMVRKHAS---TVQRTAEP--TFERKPATTDTNLINTIKLELEVKQLKERLNATEKD


*Hs_*DZIP1L LEAQREAER-----------------------------QRQLQEAELIHQREIEAKKEFD
*T. castaneum*  IQKEHQEID-----------------------------SNISRETDFKKIEDLQQKFEDL
*D. melanogaster*  IKERSTGS----------------KRVSPRQ-----EQRHVGIQSNLAEPKEKDEDSGEA
*Ae. aegypti*  LMDQRSRDREHCCHVTCIEDRGKDKDWRPPK---VVECCSVAIQSNLEDAKDVNEKEVQT
*Cx. quinquef.*  LMDQRSRD--HRCHS-CEKDRPKEREQPEQSKQVPIEVKSIAIQSNLEDFKDVNEKEVQT
*An. gambiae*  LHLHRSKH--HRCRV-CSEDSSSATDRPPAK-----VLHSVAIQSNLTDDKDSNDEKVAQ


*Hs_*DZIP1L KWKEQE------------------------WTKLYGEIDKLKKLFWDEFKNVAKQNSTLE
*T. castaneum*  RLQVQS------------------------ELKVLQTQHNFQEKYEKWFEKMALQ-GGSL
*D. melanogaster*  RQSEAS-----------------------ERKEQLTGLAERLSNFEEWQTQLKQSNEQFI
*Ae. aegypti*  TLPPVVHSPPPQPR---------VPLDYISKSELESIVREQNDQFENWKQAERLKFNSEI
*Cx. quinquef.*  SIRSKTPETPPRLSPIQRLTPSPVPLDYISKSDLEAIVREQKEQFEAWKTLEREKFNGEI
*An. gambiae*  TQTETMPQSEESATDEQR-KERAPAGDFISKSDLQLFLDEQRRLFESWKTGERQTLNQEI


*Hs_*DZIP1L EKLRALQSHSVMESKLG----SLRDEESEEWLRQARELQALREKTEIQKTEWKRKVKELH
*T. castaneum*  GRRRESMTQTEEGASSR------VVMYQEKNKENVPELSNAQKQITQFEEALETKVASSL
*D. melanogaster*  QDINKRLEGLSHALEQS-KQASASTPPLEDRVATPCLEDLERILTEKVAEIGKVSAHRLE
*Ae. aegypti*  EKVRQNLTDAIRELEKRDRMVAAAPVINVTAERNEENENIWKKRYRELEQMYENSQRQVR
*Cx. quinquef.*  EQVRRNLTAAIHELEKR-----PASIPEQRHVHDEEDDRIWKQRYHELERMYENSQRQVR
*An. gambiae*  ETVKRNLVDVIQTIEKS-------ERNTPTPVVVDESGGIWKERYHELEKIYETSQRQAQ


*Hs_*DZIP1L EEHMAEKKELQEENQRLQASLSQDQKKAAAQSQCQIST----------------------
*T. castaneum*  QKIEDQMQSFWNKLNEMEMYRCRDMEDKPKPSAKPRSK----------------------
*D. melanogaster*  EVVYHLEEGYKEKLGALERELKQLSVQKVQPEPVQTVP----------------------
*Ae. aegypti*  ETVETIESAYEEKFRRIEQMVERQKETTANEAKQDQTKRVIND---SSLLQIPVPIIDLK
*Cx. quinquef.*  ETVQNVEAVYEDKFKQLERRMERKE--AAVETRREESK----------------------
*An. gambiae*  EAIMSFEKVYTQKVEQLEKLLREARAIERKEIQNETSTGHTDLGTPHSLTHLKTVTLPTV


*Hs_*DZIP1L --LRAQLQEQARIIASQEEMIQSLSLRKVEGIHKVPKAVDTEEDSPEEEMEDSQDEQHKV
*T. castaneum*  ----YSDKHLIETRQRNESSQQRDKKQAKSVSLEEDNEITDASPQSQETPKASPIKVMRA
*D. melanogaster*  --VASKIPKPVVRKEETNIDRIRKQVESEFLKQKHDDDTYSIEEAPRKGSEKPFPQLVTQ
*Ae. aegypti*  SSLPPALSRTTFEYKVNEVEAQQDSTPDETDQSESDEEIRALEAAKAKLLSRSNVSAPAM
*Cx. quinquef.*  VLLPPPEQEQQVESEIDEAIEEHGSASEEAAQTESDDEIRALEAAKARFLAKIAVEPPP-
*An. gambiae*  VIESVSVAEDPVQQMENYVKHNTQTISSESEQVESDEELEDTNVETLKTHQQLAVKSSPL

*Hs_*DZIP1L LAALRRN--------------PTLLKHFRPILEDTLEEKLESMGIRKDAKGISIQTLRHL
*T. castaneum*  QPQIKVP----------KVKTEKIESGINLTEIDDDTTTSETLASEEKISDSPQEKVIKP
*D. melanogaster*  VQVVEKE--------------QPSAGSSDSNPTYTKSPREPAPNKQETKEATDVSDSLSQ
*Ae. aegypti*  EESKEKP---------IVSSLEKPLLSPKKLILNTFKTRLKQLGIDTKTKAISKEDLNAA
*Cx. quinquef.*  EVSHEVP---------QKP--ERKLVSPKKQIMNTFKTRLKSLGIDPKAKSLPKDDLNTV
*An. gambiae*  VATSQKPSQVTEAKPQAQSSSQALLISPKKQILSQFRARLKAIGVDPRSKQLFGENLNAA


*Hs_*DZIP1L ESLLRVQREQKARKFSEFLSLRGKLVKEVTSRAKERQENGAAVSQPDGQPSVKSQQSALV
*T. castaneum*  -PLKPQPVPRKRPKTSKDIVSSNIIDDELLQHFKLEIENILKAR-----LQDMGVSPDWK
*D. melanogaster*  EETENEEERSLTEEEGTDVPTSGSEAAREDPTPKTIKPSGRIIK--------SPQKPLTR
*Ae. aegypti*  TETLAERRDASKRQNRGFFITRNQLMTKVDQLARSRLGEAPKPR---TLRSKTVDQPIVR
*Cx. quinquef.*  AEAMAERRDVNRKKNRGFFITRNQLLAKVDQIAKGKIGESSKQ------PSKSEDLPVIK
*An. gambiae*  CKALADRRDVQKQKHGHFFVTRNQLLSRVEQLARAKIGDGSQTKESGQMKTTAHDKPIVM


*Hs_*DZIP1L TREAQPKTRTLQVALPSTPAEPPPPTRQSHGSHGSSLTQVSAPAPHPGLHGPSSTPPSSG
*T. castaneum*  GLPNRSFQRALEITCPNFDSIAS----QIEKDVAKKIDNKRQPKKKP-------------
*D. melanogaster*  KDARKMVNRKLMSHGFDMKSKG------ISHNSLKRVNSELTEHRNK-------------
*Ae. aegypti*  KRE--TPSQPVPKLRSTLQVSGNNPVIDILPSKLKLVNEDRPAVPKP-------------
*Cx. quinquef.*  KKE--SAPQPVPKFRSSLQTPS-----EILP--------DRPNALKT-------------
*An. gambiae*  GREKHFPSARGPVIKPRLKTSS---AMELLPSKQKLHLPDTGAVQHH-------------


*Hs_*DZIP1L PGMSTPPFSSEEDSEGDRVQRVSLQPPKVPSRMVPRPKDDWDWSDTETSEENAQPPGQGS
*T. castaneum*  KKLAVQIEIKNRQMYDTDTESEVKPVKVKEKQFSAPYSAVIAELKTVTSKIDASDKSEES
*D. melanogaster*  LKLQHPHFYATRNRIRKFVEKLCSAKFSERAEMLLKHKSPLKPMEVPGKGIPRSAISEKS
*Ae. aegypti*  RETTSNLFKTRTG---SSLLVAPEIKLTS--DDVITVHAEINPLAETLSRSPVPSARTSP
*Cx. quinquef.*  TTSNANLFKIKSVGPNSNFLSVPEVKHANNNDEILTVQAEINALP--------PSLRTSP
*An. gambiae*  ADMNVFKSKAIFTGGSSAPPYRPTIKLTD--DDIITVHADVTAMSG--QEDLTITPNPSK


*Hs_*DZIP1L GTLVQSMVKNLEKQLEAPAKKPAGGVSLFFMPNAGPQRAATPGRKPQLSEDESDLEISSL
*T. castaneum*  IKDEKRVKEDQTKGVLKSYPSVGSLTKKKVLFNLDTEEK------KELPAKDGGSTTSIT
*D. melanogaster*  EEDIASSQGEEQTDEQTDSSEQQTRSPSPQRLVSRDFKARLEEILVKPAATIRGASKSSL
*Ae. aegypti*  RPSVSDHDRHVERLLDTPIKRLS---TSPDVITVESSRR------AIENDSDLSDILEAV
*Cx. quinquef.*  RASFSEYERHLERLLDTPIKQVG---TPPDLITVEGRKE------ADQSDS--SDLNAA-
*An. gambiae*  RASISSYDQQVERLLHTPIKTIHPAAKTPDRVQDSTVEE------TIPSSN--NETIDSV


*Hs_*DZIP1L EDLPLDLDQREKPKPLSRSKLPEKFG----------------------------TGPQSS
*T. castaneum*  SSVFDGTPRNEEPKKKGK-------------------------------------DIEDL
*D. melanogaster*  SSRPVPLPRKRVMFNTTEDGKS-----------------------------------FND
*Ae. aegypti*  PLQPKPIPKKRVLFNLDRDNAGGAVEKAESTLNVTKEIGPKLS-----STVIQVSKAEED
*Cx. quinquef.*  ----KPVPRKRVLFNLDKQGSG-------TTDAVP--IGPTLS-----STVIHVSKADEE
*An. gambiae*  S-TLKPIPKKRVLFNLDKEDYTRTVGTSVVSAGHPGPAAEEYARSHQKPTAHSVSKVDDE


*Hs_*DZIP1L GQPRVPAW----
*T. castaneum*  SDFDFSDM----
*D. melanogaster*  SDDNLK------
*Ae. aegypti*  SDWNISSFDDEK
*Cx. quinquef.*  SDWNISSFEDEK
*An. gambiae*  SDWNISSFDEDK

**Supplementary Fig. 3**. Amino acid sequence alignment of the *Ams* putative orthologs. An entire coding sequence of the *An. gambiae* protein is presented. Since the complete gene models from culicines have not been created, the comparison is based on a highly conserved sequence region, for which the expression in *Aedes* and *Culex* was experimentally confirmed. Two sequences presented for *Aedes* are conceptual translations of transcripts strongly expressed in adult females and differing in size due to an intron splicing/retention (see Fig. 2); intron retention results in a transcript encoding a truncated protein (denoted as *Ae. aegypti*_tr in the alignment). In *Culex* pupae and adults a single transcript with an intron retained was expressed; similarly to *Aedes*, it encodes a truncated protein (denoted as *Cx. quinquef*_tr in the alignment). A second *Culex* amino acid sequence corresponds to a conceptual translation of a hypothetical transcript with the intron spliced out.

*Ae. aegypti*_tr ------------------------------------------------------------
*Ae. aegypti*  ------------------------------------------------------------
*Cx. quinquef.* ------------------------------------------------------------
*Cx. quinquef.*_tr ------------------------------------------------------------
*An. gambiae*  MLASNLQPTKNPSVGDVFRAIPSPYSAKPAQEMGHFIREVRKLPRNNTFISLEAAKEESV


*Ae. aegypti*_tr ------------------------------------------------------------
*Ae. aegypti*  ------------------------------------------------------------
*Cx. quinquef.* ------------------------------------------------------------
*Cx. quinquef._tr* ------------------------------------------------------------
*An. gambiae*  GGGKVSLQRWNSVENLTSDRQTTREQEEDKIAPIVAVKLEQFNNGSDSNDDDSSETKERN


*Ae. aegypti*_tr --PVKTEAFIGRPIRSQRSIISGSDSKTIKFLLKLTTKSYFETLKYEIDRIKEN-DRKIQ
*Ae. aegypti*  --PVKTEAFIGRPIRSQRSIISGSDSKTIKFLLKLTTKSYFETLKYEIDRIKEN-DRKIQ
*Cx. quinquef.* --PVKTNSSLGRPLRSQRSVINGSDSKTIRNLLKLITRSYFESLKHEIDRMKDN-DRRLQ
*Cx. quinquef._tr* --PVKTNSSLGRPLRSQRSVINGSDSKTIRNLLKLITRSYFESLKHEIDRMKDN-DRRLQ
*An. gambiae*  AADRRCSMIPLMAHPAGRPKVVGQPYADKEKIVRKKPPLPPKALAKNQDPVRMYRAERLQ


*Ae. aegypti*_tr DQASSNAQSIMVLINKGADFDRKMEDLRAQNVKLLLETREKDRVQLDTVNAITSELNELK
*Ae. aegypti*  DQASSNAQSIMVLINKGADFDRKMEDLRAQNVKLLLETREKDRVQLDTVNAITSELNELK
*Cx. quinquef.* ELAGSHGNSIVALISRDAEYDRRIEELREQNVRLLIETREKDKAQQTTVNTITAELNELK
*Cx. quinquef._tr* ELAGSHGNSIVALISRDAEYDRRIEELREQNVRLLIETREKDKAQQTTVNTITAELNELK
*An. gambiae*  EKKLDMEQQVDEMREQLASLELSRQKEEQRNREIIDRLKEDISDLKLTCEKLKGAVERLQ


*Ae. aegypti*_tr *-----------------------------------------------------------
*Ae. aegypti*  SVCNLLLKNIEKDRELDLLARKLHKSKRFDSNTYKMLRSQLRKP----------------
*Cx. quinquef.* SVCNLLLKNIEKDYELDLLARKLRKSKQFDSNTYKMLKTQLRKP----------------
*Cx. quinquef._tr* *-----------------------------------------------------------
*An. gambiae*  NADDLFGK-MKHEGEFSFYARSYEVSKKILSNTFRRRKANTARPSCLDVNGAETNRDTVQ


*Ae. aegypti*_tr ------------------------------------------------------------
*Ae. aegypti*  ------------------------------------------------------------
*Cx. quinquef.* ------------------------------------------------------------
*Cx. quinquef._tr* ------------------------------------------------------------
*An. gambiae*  SFNDGDQQCMLSDTAGSNSLAVMHPVGVPSGGDGDHNIRRVNSAPPETPVNIVTSMYYVS


*Ae. aegypti*_tr -------------------------------------------------
*Ae. aegypti*  -------------------------------------------------
*Cx. quinquef.* -------------------------------------------------
*Cx. quinquef._tr* -------------------------------------------------
*An. gambiae*  KIESVGMDGIPSDANDSENPMIQTPEKETMTKKKTKASKFGGWFRSGKQ

**Supplementary Fig. 4**. Amino acid sequence alignment of the *mts* orthologs.

*Ae. aegypti*  MAANSGVKIFRDILRDSPCRMAYMMSQSKNFTQQYSKNHHLGKSIPLERPGTARPKTAPI
*Cx. quinquef.* MATNTGVRFVREFLRDSPSRAAYAVVQ-RSFQQKPRR-------IPSNK---FRPQTAPI
*An. gambiae*  MAANVGSKLFRDFFQDSPCRMAYLLKSPDVSVQ-----------CPLPSKIVERPKTAPN


*Ae. aegypti*  AASSLAHAITAHNLMPETVMKPAERPTTS-VRTVKPSINLICTGTRKPDGKPSRRPEA-P
*Cx. quinquef.* CPSNLSRAVTAHKLLDDGSSGPRRRPTTS-GRPLKP--------TRVPS-KPHHRPED-A
*An. gambiae*  PLLNRTAISPVGRAPPRPMTRFDRRVQPEEFRVSAPLMLKKSPSTSGSKTKHKQSPDTKP


*Ae. aegypti*  PRQRPRRPADQSSRKTPPASNSKQDIRKRPHQQSSKLESSNTNRDSPKRPCKICISGEDC
*Cx. quinquef.* RRKAPSVPT--SSTTSLRKRPQQRQVNNKPHQQSGKPQ---------VKPCKICHEARPA
*An. gambiae*  PRKEPAKKHRTTHTTPSSGSAGGAKILNTTQKRDPQPR---------KPGCKVCLP----


*Ae. aegypti*  SVPEQWQDLLNEYSTSSSSPSSDGFRHLIGEIEAIRSELVAREDMKNISLQDSLELINEI
*Cx. quinquef.* PS-NRWPNLLANVSQ----PKNE-FHALISKIENIRAELRAQSGGH---LRDSLQLINEI
*An. gambiae*  ---------------------DDPTGFLVSEIDHIRERILGRPDSIS--CRQAIALAQDI


*Ae. aegypti*  QQRINSVSLSNGTSEGSFLNRLEPFQTATLRSEKRSTIKARKSVRSLHFEGGRIIEEDYT
*Cx. quinquef.* RQRMDLP--------ASFLDRLEPGQTQRLPS--RTELRNRKSSKSLHFEGGRIAGKDYA
*An. gambiae*  RNRVDAIVLPAG-SGGERETRIKFIGGRVESFGKLPDLKRKPSPDVVDRSXKRMKLILDK


*Ae. aegypti*  KRSRQPDGGRAV--VRKAKT----KQIEKKFDEGPLR-CLLPLVS-DSLKATRIKF
*Cx. quinquef.* TALRK-DTKKAVGSVTKFKLSCVGKPSRKQIDDGPLRKLMVPLLGRKEFSERRAIF
*An. gambiae*  KATSSPLGGWMR------------HEEERKGFKNYRRVAFFSKLCTSEIVMTHFK-

**Supplementary Fig. 5**. Amino acid sequence alignment of the *AAms* orthologs. A double-dotted line above the alignment represents a sequence region spliced as an intron in the shorter *An. gambiae* transcript*.*

*Ae. aegypti* MGRPLNRSHPTRRAIRTLSNHHLVIEVSEDGHVQVVSQSIRSMSSTDDPPELTTSIVNAP
*Cx. quinquef.* ---------MSRRRQPRSSDTQLFIDVVDDKVVKVVSE----RPVQYDPHQPVIQRLITT
*An. gambiae* --------MQNNRPKPRESPSAHERESIYDETRHMVNN---------------------L


*Ae. aegypti* PLHPTIFDGTYHYDDEDVNSEWTVSSE---SIPSETSDSEMALEEVVSKQVCSVPVIEP-
*Cx. quinquef.* PLAPTLYDGTFRHDEDGSESELSFSSDSASSVESGSGESPASVDELTLEQTGARPMFSV-
*An. gambiae* PISPTMYDGTYPAYEEFTSSLVLQSSVSELSFSSESPDSGSHVHFQNVDPIIELPSTPLK


*Ae. aegypti* SGERVEARKKSISVVGNFRGQAVGTMIDPDSTVLMQLSRGIFGGYNEILDCMADVDEMLN
*Cx. quinquef.* ARMAEEERKQSISVVSKVRKHAVGAIVDPDSTVLMQLGRKDSFGHQQALEYIRDINEMLE
*An. gambiae* DGSRQPEECTVCTDTETISQSTIGAALEKSGTVLMQRSLEN-----RLAQQLEQMHAMMG


*Ae. aegypti* NQQSVVILKYPDGNDSQRSTRTPKSHVETAKNMMEMTEDILRRVQGSSRESILVKLQNTL
*Cx. quinquef.* RDESVVILRYPNVQEEQSSRIAREDHVVTTRRMMELTEDVLGRVQGNSRESILMRLQQTL
*An. gambiae* NEDELVVLKQHSIRAMASQPAFTKDMLRDFMEHSETMFNLVHETNGNS-AQFVIGIEEAE


*Ae. aegypti* GEILRQPEDTIAIHTFSRED--TEHPRSEAFIGYFKKDDDAALFVQFHESNNEIVKSFSG
*Cx. quinquef.* EEILNQDDETIVVHSYSRADTARDLPESEALVGFFQKDDDAALFVHVPDTLNRLSSSWSG
*An. gambiae* ASDPDVPGCSHRPVRRSIVPSVTNDKGENEFVGCFSKDNDMVLYKRTMPEKPKMER----


*Ae. aegypti* CE-DEEQMGVERLKSFLQESLHSQEQILGRTLEIEPEITISRSGNTVIGVLSFPNTSSAL
*Cx. quinquef.* SETDQQQMSLDQLKNFLQESLVFQEETMGVTMSTEPDITVSKSGNRIVAVMSFPNTSSAV
*An. gambiae* ----EDRESIDSLIAHHAQCLVEADKVTSPTVHVR-----QDNDDNMLALLTFSDQSSAV


*Ae. aegypti* VRTNVDNFPVEQERSEMVRSMKDLLQNLVQQSSGKLLVQMHSESDRRTLVDQIKSELDES
*Cx. quinquef.* LTAPSASIPLESDRSEIIQAVKDRFREVIEQPSGKLLVQLHLDESRGNKVEQMKYGLDQI
*An. gambiae* LKTTSAMTEGITSEDELNEAILSTYRQLVAKPDE--MISMHTQTTPVSGQQQPAFDLDRP


*Ae. aegypti* IKSGTAESEVSAISVRESDDKVIGVLSYSGGSIVIQTSSKNWIHAHSSSDPKDLLASMRL
*Cx. quinquef.* LRSSELPHDPSQFSVQTIEDKVIGKLSYSGGSIVIQTSSKNWARPRCHK-PEDLVQSIRG
*An. gambiae* APAHRHQLRPGVMNNLSNATSSFGLSNESTFPMVVQGSSKNWIKCPRMQHAAEVVLNLRL


*Ae. aegypti* MIELFIMS-SELTDVDLRSLASIIVQAFHSARVQISDEFRNQHVLQKIKFVIHQLMNPSS
*Cx. quinquef.* VIEQFMIN-NELNDRDLKTIAALLVQVFYVEDVELPQDFVGLSVHEKIEVLVQQLMNPTV
*An. gambiae* LLEEFILRNISVTGTVLKFVSHVLSEVMRCTRQEVGDEFRRLGLVDKIKHVHNELLMPHS


*Ae. aegypti* REDPLLIINQIRNTMEDIASPEFAAG-PSEKPYSTDVLINYLDELICEENTQYDD-ALIL
*Cx. quinquef.* DEDHLRILGQIQVTLQDICQTQTGD----RDDASWDLLKSYFEGLICEGDNEYYG-DTIL
*An. gambiae* LEDPVTIVNQLRHTIEEIQNYPLKARSLASGEEAWNRVLQTLEKLSCELDHEIEHPEQFI


*Ae. aegypti* RTLRGALLDVLIHYDVSLSAALEELLEVIKMVPAENVELHPPSTVVSSPGMINIANEPFL
*Cx. quinquef.* PVLRRGLLMILSQYENSLSSALEELVQLLKQVPNEKVELYNVPSIATCPGTTTLSEP--N
*An. gambiae* EALQYGLVRVSETCTGSCS------------VPEKKVVSIDPTPVATGPAVFPTSSS---

 :::::::::::::::::::::
*Ae. aegypti* LERFSSDHPSREVVQKIHNLLGDEEPSEGREVSYLTTFQTLFLTLLRIIGGWSLRVKSFL
*Cx. quinquef.* QNAPELDHPSLEVVQKIHNLLTTDQPS---TISYLSTFHALFFAFLTIVSGWTVRVKSFL
*An. gambiae* ----------------VLEIDRYEAPSVSFCKPYTCFFERIVLSIWSVLVWIMNQMNSFW

 ::::::::::::::::::::::::::::::::::::::::::::::::::::::::::::
*Ae. aegypti* SKESRQTASTKQESESKLEPEPGTSIQRKPSVSFHLLKHPNDADERTPSNISQQLEDERQ
*Cx. quinquef.* SKDPPSPSAPPLDSQP--------SVSKKPSVSFHLVQEP--EEPSTPPDFPAQLEREQR
*An. gambiae* DFLNSPPPPLLG--------------TPSPSVSFHRSVLD-------NEKNPHQRHEESN

 ::::::::::::::::::::::::::::::::::::::::::::::::::::::::::::
*Ae. aegypti* KLNSAIDDLRTFLEESLNVPDAWDRQRKGSRLEFSGGR---TAVKQRMLHLFSKLLDEAG
*Cx. quinquef.* HLNETIDSLRAYLERTLEAPKSWQHSGQGSLVEEPEPKKLPTSGPDRMVQLFTQLLEQSE
*An. gambiae* GLNQARNVLQIALERSSGAEITADSENDPD-----------RSVNARSLKMFTQLLQDAG

 :::::::::::::::::::::
*Ae. aegypti* KLKVTEKS---CDSIHIHLDS-ASGELERKEGAQYVVLSGNVRDRQHNLGLFFEARMMDL
*Cx. quinquef.* QLRGEENPSTNANTIHLRMES-ASAVLERKEADQVVMLSGCIRDRDQNVGLFFEARLMDL
*An. gambiae* SIELKHSSS--RHNIHLQLHTSVSGMLEKREADDYLTMTGNIRDHDAGVAVFFDARIIDL

*Ae. aegypti* EEASKVESEVECVTEVRRLSDES-EELDASELVPEIVNSCLAEVGSEREEDVEQEGGGVG
*Cx. quinquef.* LGVAKQESELECVMGVRRLSDDDGQEEAAAGVVKEVLEGCLSGVVRGTDELER-------
*An. gambiae* DSLAKVESVMECVTRIGRLSDDV---ENSEVLSEETVVHSAAGTEPTTE-----------


*Ae. aegypti* EFRLQETLSRYFELIQSYMQETMDPMREALGFILEKLAAAGT------------------
*Cx. quinquef.* KTKLEQEWRRYFEQIQNHIEVSIDPLRDALEQILEKLGASKTGFEDDYFVELAETVQSEP
*An. gambiae* -------------------------DEQVVSSLIEDMVCNGG------------------


*Ae. aegypti* ----EKIREIRSSFIQTSSNFEQSYIVEEQDSREEVVEARVEDLTQQATSVQCVASVAHQ
*Cx. quinquef.* CKAIAEEVIYASKSVQFEESTKSASSVPSVRSTSDRLMTIEGDLRELKQLMQLLLASRQY
*An. gambiae* --------------------YDEELPILNTTGAEQLLREIKHCLQDLLSPVTSAVHKLCD


*Ae. aegypti* SVQNDELLIGQPSRLDNIESSLNELTQQMSELCR----------------LLRKPITEDL
*Cx. quinquef.* ELMTPEASMDSAAAEQIIMSTLDGIGWFPSQESESDLETLYASALECTDEQLAELQREEL
*An. gambiae* QFGNAGIGTAHTMAEENAS-----------------------------------------


*Ae. aegypti* PLVPPYLDSPPSTPTSPPPSIQSSDTETSFVSVVPTAPPHEEIDGRLSVTVEIVSIEEQR
*Cx. quinquef.* AKLSRRFETELGEVLETTRSIGTMVEQMSKTKLVRTVDVAVQLDG---MSLEELAAEEPL
*An. gambiae* ---------------------------------------------------ERYTVEIHD


*Ae. aegypti* RLSEVSGASDG----------------------HDQFQSAIECSTDAKLRASDEIAEPPE
*Cx. quinquef.* EETTVEGEVEQQRIPDVEEILEVVLEDILPELHPKDVEEAVEPIEAEVVQEREEPIEATV
*An. gambiae* DVTNEE--------------------------------EGHACTDHSCVICNRHVSTSNE


*Ae. aegypti* SCQVGSGEAPAR--ETTFTEMEQPEWFGELPRRSTACQTVESSGTQTDAEVSPDTIEVAV
*Cx. quinquef.* TLEAVQEEEPVEPATEGVEALEISEALDIIQEEAAELPTETSNEEDATSEIVLEPEELPE
*An. gambiae* TNENEIGVDSTPTLEQPAEELPLSETTGIEVDEALSPPQEQSTEHSQLAETMQELLELFH


*Ae. aegypti* QVVELEEPDVPEIQE------SSVVSEQRSS----------------LFAELGKRLSSLI
*Cx. quinquef.* VIEVTQEPIAPTLLEPAEPIPVEEISPQEESGIATPEEVEEELPKVEEPVESDQKVLEMI
*An. gambiae* ATNDR-------------------------------------------------------


*Ae. aegypti* TSIDAKPAVEDNK--------------------KRRSTRVSIVEPVASSDSEEDQPTDPI
*Cx. quinquef.* GFVEGSEVTEDQALEEPLQPEQVTDVEAQPIVEKSPPPLEEVVEAEQASVESVKQPSETE
*An. gambiae* ------------------------------------------LETIDTDISAIRN-----


*Ae. aegypti* CQTDMDDQIQLEQEQSSRRITTVSITEATESVESS-----------------SELNRSSL
*Cx. quinquef.* EVTVLREIIAVETTQEDRRPSEVSIVVIEPDYSQEEEVATAEDITDPIPVIIQSLEEPRP
*An. gambiae* ---EVQQLVAGQQQQQQQQP-----------------------------------VPERT


*Ae. aegypti* DDQSQDPQRRSTSASIVEPTTSFSETGEDHKRISR-QSKESDLVDEPFVQTRRSTSTSSR
*Cx. quinquef.* NLATEDLLQRDDSHTSTDKSVDISEQLEVGETLEASQEAPSDVPDDKQEQASEQPAQNLQ
*An. gambiae* HPPTQTERRRSQRKSLAQKLR-----------------------------------KSYE


*Ae. aegypti* DPAKMSTEQFRRSMDKLEEDALQMTEIPVR--------DGRNPKLCDVVLSYQCRQMDEI
*Cx. quinquef.* EPVAEEPKSLDKPLEPTEERISRTSFVKPSPMQEGDHATSTPRTLCELALSYQCQWAAYH
*An. gambiae* APIVRCPMQTQQPIGPFGQSGS--------------------------------------


*Ae. aegypti* G---RRFTSPVEIYSCHAVGPSQLMVHWKVAP-QFEDQIGGFEIYVDGEASMFYYSHRRR
*Cx. quinquef.* KGGSQRFACPAGIISCHRVGPDQLMLHWDVAA-KYLDQIDDFKIYVDGQEHRGYFSSKRR
*An. gambiae* -------YCPTEIYSCHAVSPDVLVVHWRVLDDDVLHCIAGFEVYVDNELRSVCYSNKRR


*Ae. aegypti* TGLLEDIDTEKQHRIVIYCNPKSW---ISDG-VQWAPGIFFYHL
*Cx. quinquef.* RTLISEINTAGEHLISIYPTPKKE---VSGGNLQWAPGFFVYHQ
*An. gambiae* TTLIGNIDLKKHHQITLHITTRDDSGSACEKAAQWAPAFFLYHT
